# The Study of Hypoglycemic Activity of 7-Terpenylcoumarins

**DOI:** 10.3390/molecules27248663

**Published:** 2022-12-07

**Authors:** Sergey Kuranov, Mariya Marenina, Dmitriy Ivankin, Mikhail Blokhin, Sergey Borisov, Tatyana Khomenko, Olga Luzina, Mikhail Khvostov, Konstantin Volcho, Tatyana Tolstikova, Nariman Salakhutdinov

**Affiliations:** N. N. Vorozhtsov Novosibirsk Institute of Organic Chemistry, 9, Akademika Lavrentieva Ave., Novosibirsk 630090, Russia

**Keywords:** terpene, coumarins, diabetes mellitus, hypoglycemic activity, OGTT, DPP IV

## Abstract

Natural and synthetic coumarins are often considered privileged scaffolds for obtaining pharmacological agents with hypoglycemic activity. Chemical modification of coumarins often leads to antidiabetic agents with greater efficacy. In the present work, twenty monoterpene-substituted 7-hydroxycoumarins were synthesized. A new approach using the Mitsunobu reaction was shown to be effective for the synthesis of target compounds. All of the synthesized compounds were evaluated in an oral glucose tolerance test, and two of them containing geranyl and (-)-myrtenyl substituents showed in vivo hypoglycemic action. A possible mechanism of action of these compounds may include inhibition of DPP IV, which was proved in an in vitro test.

## 1. Introduction

Natural and synthetic coumarins (2*H*-chromene-2-one) exhibit a variety of biological activities and are often considered privileged scaffolds for obtaining more active pharmacological agents on their basis [1]. The hypoglycemic activity of coumarins is widely known [2]. Among the most typical hypoglycemic targets of coumarins, the most common are protein-tyrosine phosphatase 1B (PTP1B) [3], Dipeptidyl peptidase-4 (DPP-IV) [4,5] and alpha-glycosidase [6].

Chemical modification of coumarins’ backbone leading to the creation of hybride structures is one of the common tools for expanding coumarin libraries [7]. In addition, it has been shown that chemical modification of coumarins often leads to antidiabetic agents with greater efficacy in comparison with parent molecules [8]. The localization of the chemical modification and the type of the inserted substituent greatly affects some aspects of the antidiabetic action. For example, among the 3-arylcoumarin derivatives, compounds with hydroxyl at position 7 or with hydroxyl groups at 5 and 7 positions and 4′-OH substituent had significant inhibitory activity against α-glucosidase; catechol derivatives, 4-methyl-7-aminocoumarins and dicoumarins have marked antioxidant properties (effectively removing ROS); osthole delays the onset and development of diabetic neuralgia, a complication of diabetes, by counteracting P2X3 expression in diabetic rats; coumarin-chalcone hybrids have a significant effect on insulin resistance [8].

Monoterpenes and monoterpenoids have their own promising potential as antidiabetic therapy [9]. The combination of fragments of two natural compounds in one molecule, each capable of binding to hypoglycemic targets, could lead to a new type of antidiabetic agent with possibly multitarget action. However, to the best of our knowledge, the hypoglycemic activity of terpenylcoumarins has not been studied so far.

**Figure 1:**
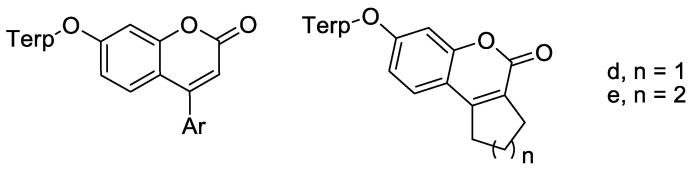
Structures of 7-terpenylcoumarins with antiviral and Tdp1-inhibiting activity.

Prenylated coumarin derivatives were found in nature and have a wide range of biological activities: antitumor, neuroprotective, anti-inflammatory, antiviral, etc. [10,11,12]. They can also be synthesized chemically. An extensive library of terpenylcoumarins was previously synthesized in our laboratory by condensation reaction of 7-hydroxycoumarins with bromine derivatives of terpenes [13,14,15]. It has been shown that such modification of coumarins leads to an increase in enzyme-inhibitory activity for 4-arylcoumarins [15] and antiviral activity for compounds containing the cycloalkane ring annulated with the coumarin framework [14] (Figure 1).

In this work, we proposed a new efficient method for the preparation of 7-terpenylcoumarins from hydroxycoumarins and terpene alcohols in one, which leads to an increase in the yield of the target terpenylcoumarins. We studied hypoglycemic activity on mice in the oral glucose tolerance test (OGTT) of a library of semi-synthetic coumarins containing terpene fragments at position 7 and modified by either introduction of aryl substituent at position 4 or annelation of cyclopentane or cyclohexane fragment at positions 3, 4. The in vitro inhibitory effect of terpenylcoumarins on DPP IV was also studied.

## 2. Results

### 2.1. Chemistry

**Scheme 1:**
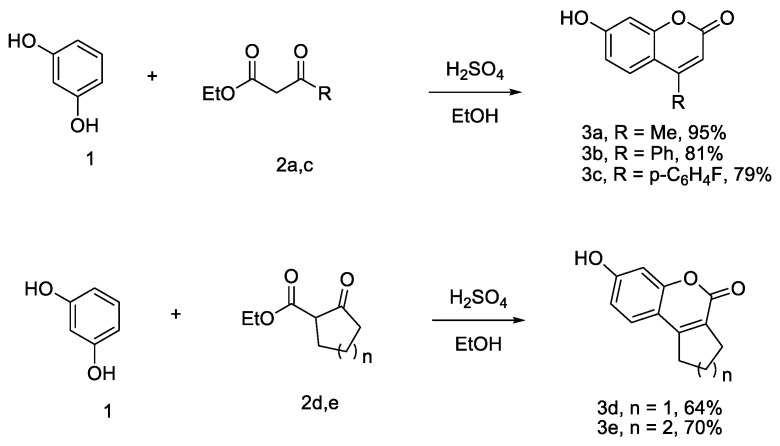
Synthesis of 7-hydroxycoumarins.

The main approach to the preparation of hydroxyl group unsubstituted hydroxycoumarins is an acid-catalyzed Pechmann condensation between resorcinol **1** and commercially available β-ketocarboxylic acid esters **2a–e** [14] (Scheme 1). Using this approach, 7-hydroxycoumarins **3a–e** were synthesized in 63–81% yields (Scheme 1).

The target terpenylcoumarins **6–10a–d** were obtained using two techniques.

**Scheme 2:**
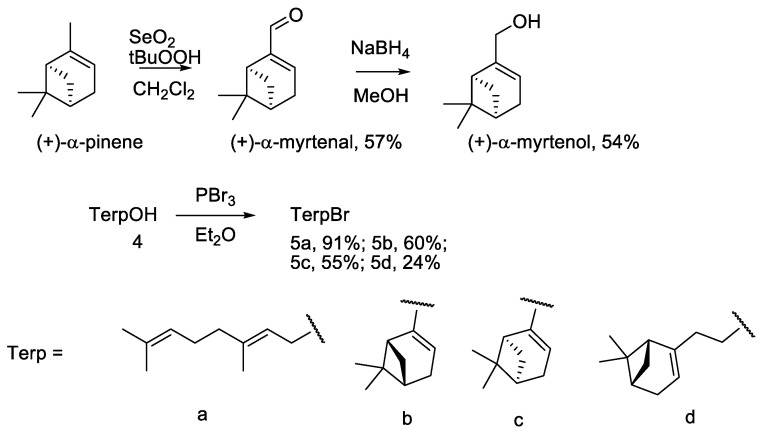
Synthesis of terpenyl bromides.

Method **A**: Compounds **6a–c**, **7a–d**, **8a–d**, **9a–d** and **10a–d** were synthesized according to [14,15] by reacting hydroxycoumarins with terpenyl bromides. Terpenyl bromides used for reaction with the phenolic group of coumarins were synthesized from commercially available alcohols which reacted with PBr_3_ in ether [13] (Scheme 2). As the starting compounds, we chose commercially available monoterpenoid alcohols: (−)-myrtenol, (−)-nopol and geraniol. Moreover, (+)-myrtenol (**4c**) was synthesized from commercially available (+)-α-pinene according to literature procedures [13].

**Scheme 3:**
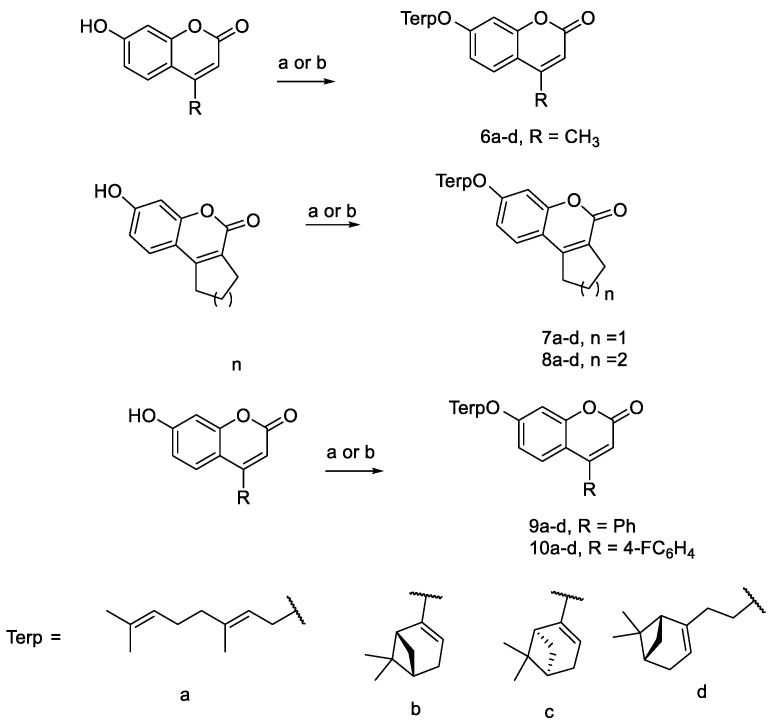
Synthesis of 7-terpenylcoumarins. Method A: **5a–d**, K_2_CO_3_, EtOH, 60 °C; method B: **4a–d**, DIAD, PPh_3_, THF, 0 °C—r.t.

Target compounds **6a–c**, **9a–d**, **10a–d**, **7a–d** and **8a–d** were prepared by reaction of coumarins **3a–e** with synthesized bromides **5a–d** according to the procedure shown in Scheme 3 (Method **A**). The products were purified by column chromatography or/and recrystallization.

Method **B**: Compounds **6a–d**, **7a**, **7c**, **7a**, **8a**, **8c**, **9c**, **9d**, **10b** and **10d** were synthesized using the Mitsunobu reaction, in which a terpene alcohol reacts directly with the hydroxycoumarin. According to the procedure shown in Scheme 3 (method **B**), terpenylcoumarins were synthesized via the reaction of 7-hydroxycoumarins **3a–e**, alcohols **4a–d**, diisopropyl azodicarboxylate (DIAD) and triphenylphosphine in dry tetrahydrofuran. The products were purified by column chromatography and isolated with the yields of 47–71% (Table 1).

The Table 1 shows comparative data on the yields of 7-terpenylcoumarins obtained by methods **A** or **B**.

### 2.2. The OGTT in C57BL/6 Mice

**Figure 2:**
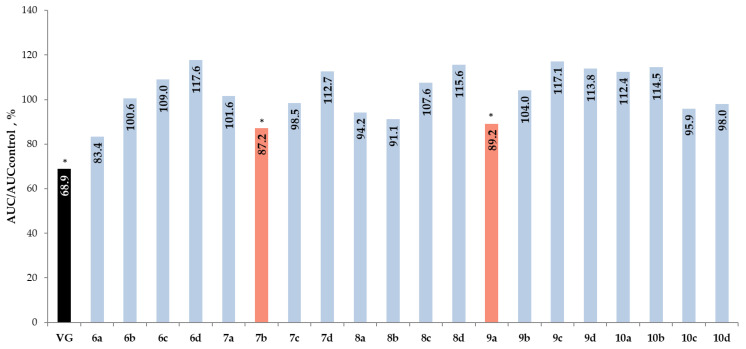
Results of the OGTT. Data are presented as the percent of the AUC/AUC control ratio, where AUC is the area under the glycemic curve of the tested compound group, and AUC control is the area under the glycemic curve of the respective control group. Doses: 10 mg/kg (VG, **6a**–**7c**, **8a**–**8c**) and 15 mg/kg (**7d**, **8d**–**10d**); *—compounds whose AUC was significantly (*p* ≤ 0.05) lower than the corresponding AUC control; VG—Vildagliptin.

An OGTT in C57BL/6 mice (*n* = 6 per each group) was performed in order to assess the hypoglycemic activity of all obtained compounds. Doses of the substances tested were calculated taking into account their molecular weight, so that the number of molecules corresponded to that of vildagliptin taken as positive control. According to the results of the area under the glycemic curve (AUC) calculations, it was found that compared to the control group, three compounds showed a moderate reduction (under 90%) in blood glucose levels (**6a**, **7b** and **9a**) (Figure 2).

Yet, the results of the statistical analysis show that only the AUC of compounds **7b** and **9a** was significantly lower than the AUC of the corresponding control group (Figure S23), while the **6a**, despite its AUC/AUC_control_ being the lowest among all tested compounds, did not show any statistically significant results (Figure S24).

### 2.3. The In Vitro DPP IV Assay

**Figure 3:**
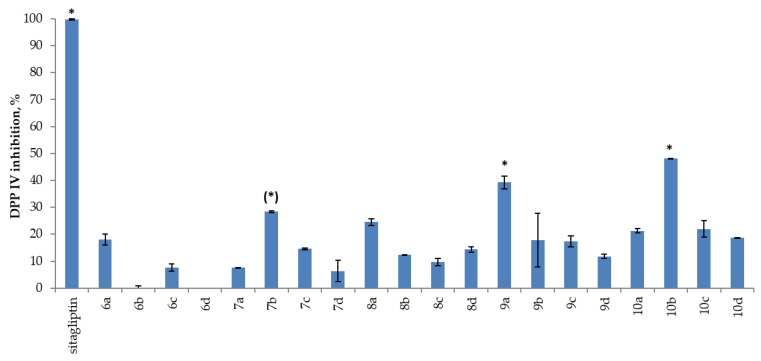
DPP IV inhibition (%) by all synthesized compounds at 100 μM. Measurements were performed in duplicate. * *p* < 0.05, (*) 0.05 < *p* < 0.1 as compared to control.

**Figure 4:**
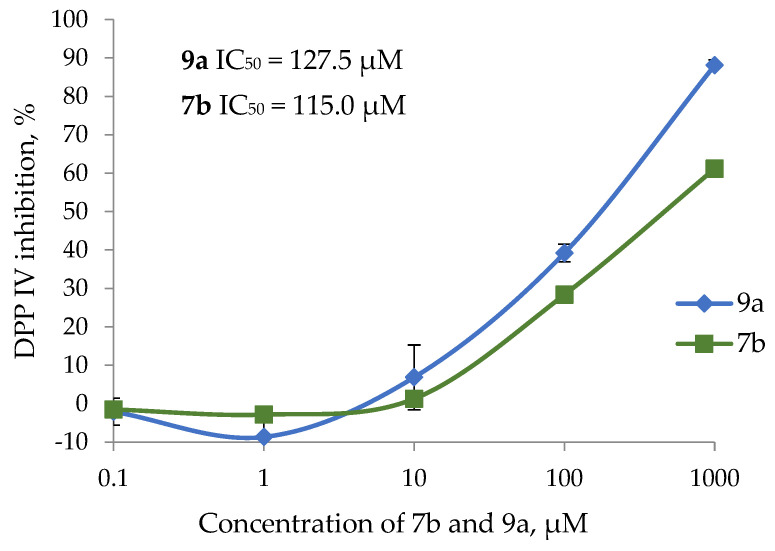
DPP IV inhibition (%) by **7b** and **9a** at concentration 0.1–1000 μM. Measurements were performed in duplicate. IC_50_ was calculated using a four-parameter logistic regression model.

Synthesized compounds were tested for their ability to inhibit DPP IV at a concentration of 100 μM. Most of the compounds showed weak inhibitory activity. Moderate (20–40%) activity was observed for a number of derivatives (Figure 3). Compounds **9a** and **10b** demonstrated statistically significant inhibition, while the effect of the **7b** was found as a tendency. We determined their IC_50_ value for the DPP IV inhibition, which exceeds 100 μM for both compounds (127.5 μM for **9a**, 115 μM for **7b**) (Figure 4). The IC_50_ value for other compounds that were close in DPP IV inhibition to **7b** and **9a** was not determined since they did not show action in vivo.

## 3. Discussion

The method for the synthesis of 7-terpenylcoumarins described in the literature includes a stage of the synthesis of terpenyl bromides and their subsequent condensation with 7-hydroxycoumarins. Since the terpenylbromides are usually unstable, often obtained in low yields (the yield of nopylbromide 24%), we proposed an alternative approach to the synthesis of terpenylcoumarins using the Mitsunobu reaction, in which a terpene alcohol reacts directly with the hydroxycoumarin. This approach avoids the need to use terpene bromides and also increases the total yield. Moreover, using this approach, we synthesized coumarin **6d**, previously not available and not described in the literature, with a yield of 50%. The table shows comparative data on the yields of 7-terpenylcoumarins obtained by methods A and B. The yields of the target compounds using the Mitsunobu reaction are in all cases higher or significantly higher (compounds **6a–d**, **7a**, **7c**, **7a**, **8a**, **8c**, **9c**, **9d**, **10b**, **10d**) than those reached using method A.

Twenty 7-terpenylcoumarins differing in the structures of both the terpene fragment and the coumarin moiety were tested in the OGTT test. According to the results of the AUC calculations, it was found that, although not reaching the level of the hypoglycemic action of the reference drug Vildagliptin at a dose of 10 mg/kg, compounds **7b** at a dose of 10 mg/kg and **9a** at a dose of 15 mg/kg demonstrated a 12.8% and 10.8% AUC level reduction compared to the control group, respectively. The AUC of all other tested agents was either almost equal to that of the control group or slightly higher (up to 17.6%). The compounds that raised the blood glucose level were considered not active, because the aim of the present study was to investigate the hypoglycemic activity of the tested substances.

Analyzing the OGTT data, we noted that only coumarins containing the geranyl and (−)-myrtene substituents had a hypoglycemic effect on mice. The structure of the terpene residue turned out to be the most important factor influencing the studied effect. Thus, replacement of the (−)-myrtene substituent with its optical antipode ((+)-myrtene) resulted in loss of activity (compare compounds **7b** and **7c**). The lengthening of the alkyl chain connecting the (−)-pinene fragment to the coumarin backbone by one methylene group led to the same results (compare compounds **7b** and **7d**). Within the group of compounds having the same terpene substituent, we can trace the effect of the coumarin backbone structure on the activity. Thus, among the (−)-myrtenyl derivatives (compounds **6b–10b**), only compounds **7b** and **8b** with annelated cyclopentane and cyclohexane rings exhibit hypoglycemic activity. Replacement of cyclic fragments with a methyl or aryl substituent at position 4 leads to loss of activity. Conversely, in the group of geranyl derivatives (compounds **6a–10a**), coumarins with alkyl substituents (annelated cycloalkyl fragment or 4-methyl) are inactive, whereas 4-phenyl substituent (compound **9a**) has hypoglycemic effect. Introduction of fluorine, often resulting in enhanced pharmacological action [16], in our case was not affected at all.

Most of the compounds showed weak inhibitory activity on DPP IV in vitro. Moderate (20–40%) activity was observed for a number of derivatives at 100 μM. Compounds **9a** and **10b** demonstrated statistically significant inhibition while the effect of the **7b** was found as a tendency. Among them, two compounds (**7b**, **9a**) showed a statistically significant effect on the OGTT IC_50_ value, both compounds exceeding 100 μM (127.5 μM for **9a**, 115 μM for **7b**) (Figure 4).

It is worth noting that in the series of synthesized compounds, the highest DPP IV inhibitory activity was demonstrated by compounds whose statistically significant hypoglycemic activity was found in animal tests. For these compounds (**7b**, **9a**), there was a correlation between the degree of DPP IV inhibition and the level of glucose reduction in OGTT, namely moderate enzyme inhibition and moderate hypoglycemic action.

However, it should be noted that compound **10b**, which showed a higher level of inhibitory activity, did not show a significant hypoglycemic effect in OHTT, which may indicate the low bioavailability of this compound or the presence of other mechanisms to reduce blood glucose levels in compounds **7b** and **9a**.

The ability to inhibit DPP IV for geranyl-containing compounds has previously been shown on the example of geranyl-substituted stilbenoids. Their IC_50_ was significantly lower than that of our substances and did not exceed 22 μM [17]. Geraniol itself also exhibits hypoglycemic action, but this effect is observed in doses greater than 100 mg/kg, and its mechanism of action includes effects on the enzymes of glycolysis and gluconeogenesis and a protective effect on the pancreatic islet apparatus of rats [18]. No direct hypoglycemic action has been described for myrtenol. It is known that it can reduce the severity of streptozocin-induced diabetes in rats by reducing oxidative stress and inflammation [19]. Thus, the hypoglycemic effect of mirtenol-containing molecules was shown for the first time.

## 4. Materials and Methods

### 4.1. Chemistry

^1^H and ^13^C NMR spectra were acquired on Bruker spectrometers AV-400 at 400.13 MHz (^1^H) and 100.61 MHz (^13^C), AV-600 at 600.30 MHz (^1^H) and 150.95 MHz (^13^C) in CDCl_3_ or DMSO-d_6_; chemical shifts δ in ppm were measured relative to residual CHCl_3_ (δ(CHCl_3_) 7.26, δ(CHCl_3_) 77.00 ppm), and *J* was measured in Hertz. The structure of all synthesized compounds except **6d** was confirmed by comparing their spectra with literature data [13,14,15]. The structure of the product **6d** was determined by means of ^1^H and ^13^C NMR spectra. Optical rotation was measured on a polar 3005 spectrometer (EtOH solution). High-resolution mass spectrometry was conducted using a DFS Thermo Scientific spectrometer in full-scan mode (15–500 m/z, 70 eV electron impact ionization and direct sample injection). Column chromatography was performed on silica gel (60–200 l, Macherey-Nagel). All the compounds reported in this paper had purity of at least 95%. Spectral and analytical measurements were carried out at the Multi-Access Chemical Service Center of SB RAS.

All reagents were analytically pure and were used as received without purification prior to use. Solvents were distilled prior to use: THF by distillation from sodium/benzophenone under nitrogen, whereas dichloromethane and acetonitrile were distilled from phosphorus pentoxide.

Commercially available compounds β-ketoesters and terpenoids were obtained from Acros (Waltham, MA, USA) and Sigma-Aldrich (St. Louis, MO, USA).

Synthesis of compound **4c**. (+)-Myrtenylbromide was synthesized according to the procedure [13].

A 70% solution of tertbutyl hydroperoxide (65 mL) was extracted with dichloromethane (82 mL). After decantation and separation of a water layer, the organic layer was stirred at room temperature with selenium dioxide (0.54 g, 4.9 mmol) until dissolution. Then, (+)-α-pinene (18.5 g, 0.14 mol) was added in a 3 h period, the temperature being kept under 30 °C. After the addition was complete, the mixture was stirred at 35 °C for 48 h. The final mixture was washed with a 10% aqueous solution of potassium hydroxide (50 mL) and then with water in order to reach a pH = 7. The organic layer was dried over Na_2_SO_4_ and the solvent removed under reduced pressure to yield an oil (25 g) containing (+)-myrtenal and used in the following step without further purification. NaBH4 (0.11 mol) was added to a cooled (0–5 °C) solution of (+)-myrtenal in methanol (125 mL), and the reaction mixture was stirred for 3 h at room temperature. Then, 5% aqueous HCl was added to reach a pH of 4–5. The solvent was distilled, and the product was extracted using ether and dried with Na_2_SO_4_. The solvent was evaporated; the rest was distilled under reduced pressure to obtain colorless syrup. The yield of 49% in terms of pinene. bp 94–100 °C (7.6 mm) [20].

Synthesis of compound **5a–d**.

Terpenylbromides **5a–d** were synthesized by the reaction terpenoalcohols **4a–d** with PBr_3_ [21].

PBr_3_ (2.5 mmol) was added to cooled (0–5 °C) solution of 7.5 mmol of appropriate alcohol in dry ether (10 mL), and the reaction mixture was stirred for 2 h at rt. Saturated aqueous NaHCO_3_ was added, and the product was extracted with ether. The extracts were washed with brine, dried with Na_2_SO_4_ and evaporated. Compounds **5a–c** (yields 91%, 55% and 60%, respectively) were sufficiently pure and used for the next step without purification. The compound **5d** was purified by column chromatography on SiO_2_ (yield 24%).

Synthesis of compounds **3a–e**.

Compounds **3a–e** were synthesized according to the procedure [22].

Conc. H_2_SO_4_ (5 mL, 94 mmol) was added dropwise to cooled (0–5 °C) solution of resorcin **1** (45 mmol) and appropriate β-keto esters (**2a–e**) (45 mmol) in dry ethanol (15 mL) with vigorous stirring. The mixture was stirred until congealed, left overnight at r.t. and poured into ice water (150 mL). The resulting solid was filtered off and crystallized from ethanol-water (3:1). Compounds **3a–e** were obtained with yields of 95, 81, 79, 64 and 70%, respectively.

Synthesis of compounds **6–10**.

Method **A**:

To 0.5 mmol of corresponding compound **3a–e** in dry ethanol (5 mL), 0.75 mmol of K_2_CO_3_ and 0.75 mmol of bromide **5a–d** were added at rt under stirring. The reaction mixture was stirred at rt for 15 min and then heated at 60 °C for 5 h. The hot solution was filtered; the filtrate was kept at −18 °C for 48 h. The products were isolated in the individual form by column chromatography on silica gel; eluent—solution containing from 25 to 100% chloroform in hexane.

(E)-7-(3,7-Dimethylocta-2,6-dienyloxy)-4-methyl-2H-chromen-2-one (**6a**). Yield 47%.

7-(((1R,5S)-6,6-Dimethylbicyclo [3.1.1]hept-2-en-2-yl)methoxy)-4-methyl-2H-chromen-2-one (**6b**). Yield 54%.

7-(((1S,5R)-6,6-Dimethylbicyclo [3.1.1]hept-2-en-2-yl)methoxy)-4-methyl-2H-chromen-2-one (**6c**). Yield 30%.

(E)-7-(3,7-Dimethylocta-2,6-dienyloxy)-2,3-dihydrocyclopenta[c]chromen-4(1H)-one (**7a**). Yield 37%.

7-(((1R,5S)-6,6-Dimethylbicyclo [3.1.1]hept-2-en-2-yl)methoxy)-2,3-dihydrocyclopenta[c]chromen-4(1H)-one (**7b**). Yield 40%.

7-(((1S,5R)-6,6-Dimethylbicyclo [3.1.1]hept-2-en-2-yl)methoxy)-2,3-dihydrocyclopenta[c]chromen-4(1H)-one (**7c**). Yield 55%.

7-(2-((1R,5S)-6,6-Dimethylbicyclo [3.1.1]hept-2-en-2-yl)ethoxy)-2,3-dihydrocyclopenta[c]chromen-4(1H)-one (**7d**). Yield 29%.

(E)-3-(3,7-Dimethylocta-2,6-dienyloxy)-7,8,9,10-tetrahydro-6H-benzo[c]chromen-6-one (**8a**). Yield 53%.

3-(((1R,5S)-6,6-Dimethylbicyclo [3.1.1]hept-2-en-2-yl)methoxy)-7,8,9,10-tetrahydro-6H-benzo[c]chromen-6-one (**8b**). Yield 38%.

3-(((1S,5R)-6,6-Dimethylbicyclo [3.1.1]hept-2-en-2-yl)methoxy)-7,8,9,10-tetrahydro-6H-benzo[c]chromen-6-one (**8c**). Yield 38%.

3-(2-((1R,5S)-6,6-Dimethylbicyclo [3.1.1]hept-2-en-2-yl)ethoxy)-7,8,9,10-tetrahydro-6H-benzo[c]chromen-6-one (**8d**). Yield 32%.

(E)-7-(3,7-Dimethylocta-2,6-dienyloxy)-4-phenyl-2H-chromen-2-one (**9a**). Yield 56%.

7-(((1R,5S)-6,6-Dimethylbicyclo [3.1.1]hept-2-en-2-yl)methoxy)-4-phenyl-2H-chromen-2-one (**9b**). Yield 40%.

7-(((1S,5R)-6,6-Dimethylbicyclo [3.1.1]hept-2-en-2-yl)methoxy)-4-phenyl-2H-chromen-2-one (**9c**). Yield 46%.

7-(2-((1R,5S)-6,6-dimethylbicyclo [3.1.1]hept-2-en-2-yl)ethoxy)-4-phenyl-2H-chromen-2-one (**9d**). Yield 12%.

(E)-7-(3,7-Dimethylocta-2,6-dienyloxy)-4-(4-fluorophenyl)-2H-chromen-2-one (**10a**).

Yield 35%

7-(((1R,5S)-6,6-Dimethylbicyclo [3.1.1]hept-2-en-2-yl)methoxy)-4-(4-fluorophenyl)-2H-chromen-2-one (**10b**). Yield 53%.

7-(((1S,5R)-6,6-Dimethylbicyclo [3.1.1]hept-2-en-2-yl)methoxy)-4-(4-fluorophenyl)-2H-chromen-2-one (**10c**). Yield 35%.

7-(2-((1R,5S)-6,6-Dimethylbicyclo [3.1.1]hept-2-en-2-yl)ethoxy)-4-(4-fluorophenyl)-2H-chromen-2-one (**10d**). Yield 37%.

Method **B**:

A total of 0.42 mmol of corresponding compound **3a–e** and 0.48 mmol of alcohol **4a–d** were dissolved in 2 mL of freshly distilled tetrahydrofuran, and the resulting solution was cooled in an ice bath. To the cooled stirred solution was gently added diisopropyl azodicarboxylate (DIAD) (0.127 mL, 0.48 mmol). The resulting solution was stirred for 2 h while cooling in an ice bath and another 48 h at room temperature. The solvent was evaporated, and the residue was chromatographed on silica gel (eluent-hexane/ethyl acetate 20:1) to result in compounds **6–10**.

(E)-7-(3,7-Dimethylocta-2,6-dienyloxy)-4-methyl-2H-chromen-2-one (**6a**). Yield 64%.

7-(((1R,5S)-6,6-Dimethylbicyclo [3.1.1]hept-2-en-2-yl)methoxy)-4-methyl-2H-chromen-2-one (**6b**). Yield 70%.

7-(((1S,5R)-6,6-Dimethylbicyclo [3.1.1]hept-2-en-2-yl)methoxy)-4-methyl-2H-chromen-2-one (**6c**). Yield 60%.

7-(2-((1R,5S)-6,6-Dimethylbicyclo [3.1.1]hept-2-en-2-yl)ethoxy)-4-methyl-2H-chromen-2-one (**6d**). White solid.Yield 50%. m.p. 112.9–114.6 °C. [α]^25^_D_-13 (c 0.327, EtOH).

^1^H-NMR (400 MHz, CDCl_3_) (ppm) δ: 0.80 (c, 3H, H-20), 1.15 (d, 1H, *J* = 8.5 Hz, H-19^a^), 1.25 (s, 3H, H-21), 2.04–2.10 (m, 2H, H-16, H-18), 2.14–2.32 (m, 2H, H-15^a^, H-19^b^), 2.33–2.40 (m, 1H, H-15^b^), 2.37 (d, 3H, *J* = 1.2 Hz, H-10), 2.41–2.50 (m, 2H, H-12), 4.00 (t, 2H, *J* = 6.8 Hz, H-11), 5.34 (m, 1H, H-14), 6.10 (q, 1H, *J* = 1.1 Hz, H-3), 6.76 (d, *J* = 2.5 Hz, H-9), 6.81 (dd, 1H, *J* = 8.8 Hz, *J* = 2.5 Hz, H-7), 7.44 (d, 1H, *J* = 8.8 Hz, H-6). ^13^C-NMR (75 MHz, CDCl_3_) (ppm) δ: 154.8 (C-1), 161.6 (C-2), 112.3 (C-3), 152.2 (C-4), 113.0 (C-5), 125.0 (C-6), 111.4(C-7), 160.9 (C-8), 100.9 (C-9), 18.2 (C-10), 66.5 (C-11), 35.7 (C-12), 143.5 (C-13), 118.7 (C-14), 30.92(C-15), 40.2 (C-16), 37.6 (C-17), 45.4 (C-18), 31.2 (C-19), 20.8 (C-20), 25.8 (C-21). HRMS for C21H23O3^+^ calcd 324.1720, found 323.1643 [M]^+^.

(E)-7-(3,7-Dimethylocta-2,6-dienyloxy)-2,3-dihydrocyclopenta[c]chromen-4(1H)-one (**7a**). Yield 71%.

7-(((1S,5R)-6,6-Dimethylbicyclo [3.1.1]hept-2-en-2-yl)methoxy)-2,3-dihydrocyclopenta[c]chromen-4(1H)-one (**7c**). Yield 56%.

(E)-3-(3,7-Dimethylocta-2,6-dienyloxy)-7,8,9,10-tetrahydro-6H-benzo[c]chromen-6-one (**8a**). Yield 69%.

3-(((1S,5R)-6,6-Dimethylbicyclo [3.1.1]hept-2-en-2-yl)methoxy)-7,8,9,10-tetrahydro-6H-benzo[c]chromen-6-one (**8c**). Yield 54%.

7-(((1S,5R)-6,6-Dimethylbicyclo [3.1.1]hept-2-en-2-yl)methoxy)-4-phenyl-2H-chromen-2-one (**9c**). Yield 58%.

7-(2-((1R,5S)-6,6-Dimethylbicyclo [3.1.1]hept-2-en-2-yl)ethoxy)-4-phenyl-2H-chromen-2-one (**9d**). Yield 60%.

7-(((1R,5S)-6,6-Dimethylbicyclo [3.1.1]hept-2-en-2-yl)methoxy)-4-(4-fluorophenyl)-2H-chromen-2-one (**10b**). Yield 70%.

7-(2-((1R,5S)-6,6-Dimethylbicyclo [3.1.1]hept-2-en-2-yl)ethoxy)-4-(4-fluorophenyl)-2H-chromen-2-one (**10d**). Yield 47%.

### 4.2. Biological Experiments

#### 4.2.1. Animals

Male C57BL/6 mice weighing 22–25 g were obtained from the specific pathogen-free vivarium of the Institute of Cytology and Genetics SB RAS. The animals were kept under standard conditions with free access to water and food in humidity-and-temperature-controlled rooms on a 12/12 h light-and-dark cycle. All manipulations with animals were carried out in strict accordance with the legislation of the Russian Federation, a decree of the Ministry of Health of the Russian Federation No. 199n of 4 January 2016 and the provisions of Directive 2010/63/EU of the European Parliament and of the Council of the European Union of 22 September 2010 on the protection of animals used for scientific purposes.

#### 4.2.2. The OGTT

The test was performed on mice after a 12 h fast (*n* = 6 in each group). Oral glucose loading (2.5 g/kg) was performed in all the groups of mice. Prior to dissolution in water, all compounds were mixed with two drops of Tween 80. Vildagliptin tablets (Galvus, Novartis) were dissolved in water. The tested compounds were given by oral gavage 30 min prior to the glucose load. Blood glucose was quantified with a ONE TOUCH Select blood glucose meter (LIFESCAN Inc., Milpitas, CA, USA) before dosing (time 0) and at 30, 60, 90 and 120 min after the glucose load. The area under the glycemic curve was calculated using Tai’s model [23].

#### 4.2.3. In Vitro Investigation

A fluorometric assay kit (DPP (IV) Inhibitor Screening Assay Kit, Cayman chemical) was used to determine the DPP-IV inhibitory activity of compounds **6–10**. Sitagliptin (as part of a kit) was used as positive control inhibitor. Substances were diluted in dimethylsulfoxide (DMSO (Reagent Component, Moscow, Russia)) and tested at a final concentration of 100 µM (in 10% DMSO). For two compounds (**7b**, **9a**), inhibitory activity was also determined at concentrations of 0.1, 1, 10 and 1000 µM to estimate IC_50_. The assay was based on liberation of AMC (7-amino-4-methyl-coumarin) from DPP-IV substrate, Gly-Pro-AMC. Changes in fluorescence due to cleavage of the molecule by DPP-IV were measured with an excitation and emission wavelength at 350 and 450 nm using a ClarioStar (BMG Labtech, Germany). Percentage inhibition of DPP-IV activity was calculated relative to baseline activity (control group, without inhibitors) using the formula: %inhibition = (baseline activity − activity with inhibitor) / baseline activity * 100. IC_50_ (for compounds **7b** and **9a**) was calculated using a four-parameter logistic regression model.

#### 4.2.4. Statistical Analyses

Statistical analysis was performed by the Mann–Whitney U test. Data are shown as mean ± SEM. Data with *p* < 0.05 were considered statistically significant.

## 5. Conclusions

Twenty 7-O-monoterpenylcoumarins differing in the structure of the terpenyl substituent and the substituent in positions 3 and 4 of the coumarin backbone were synthesized using the previously described and new approaches to the synthesis. It was shown that synthesis by the Mitsunobu reaction leads to a marked increase in the yields of the target compounds. The hypoglycemic effect of 7-O-terpenylcoumarins in the oral glucose tolerance test (OGTT) in animals was studied for the first time and their inhibitory effect on DPP IV was evaluated. The hypoglycemic effect was shown to depend both on the structure of the terpenyl substituent and on modifications of the piranone ring of the coumarin backbone. Two compounds containing the geranyl and (−)-myrtene substituents showed a significant decrease in the glucose level in the OGTT when administered at doses of 10 and 15 mg/kg, respectively. In vitro test data on DPP IV inhibition correlate with glucose tolerance test data, which may indicate the participation of DPP IV in the mechanism of hypoglycemic action of terpenylcoumarins.

## Figures and Tables

**Table 1:** Yields of compounds **6–10** (%), obtained by methods **A** (black color) or **B** (red color).

Coumarin/Terp	a	b	c	d
**6**	47/64	54/70	30/60	50
**7**	37/71	40	55/56	29
**8**	53/69	38	38/54	32
**9**	56	40	46/58	12/60
**10**	35	53/70	35	37/47

## Data Availability

Not applicable.

## Supplementary Materials

The following supporting information can be downloaded at: https://www.mdpi.com/article/10.3390/molecules27248663/s1, Figures S1–S22: NMR spectra for compounds **6**–**10**; Figures S23–S24: OGTT results.
