# Supplementary material for: The Study of Hypoglycemic Activity of 7-Terpenylcoumarins
Source: Molecules. 2022 Dec 7;27(24):8663. doi: 10.3390/molecules27248663 (PMC9785006; doi:10.3390/molecules27248663)
Supplement: Supplementary file 1 [file molecules-27-08663-s001.zip › molecules-2028468-supplementary materials.pdf]

Supporting Information for:

The study of hypoglycemic activity of 7-terpenylcoumarines

Sergey Kuranov, Mariya Marenina, Dmitriy Ivankin, Mikhail Blokhin, Sergey Borisov, Tatyana Khomenko, Olga Luzina*, Mikhail Khvostov, Konstantin Volcho, Tatyana Tolstikova, Nariman Salakhutdinov

N. N. Vorozhtsov Novosibirsk Institute of Organic Chemistry, 9, Akademika Lavrentieva Ave., Novosibirsk, 630090, Russian Federation; luzina@nioch.nsc.ru

* Correspondence: luzina@nioch.nsc.ru

INDEX

(E)-7-(3,7-Dimethylocta-2,6-dienyloxy)-4-methyl-2H-chromen-2-one ^{1}H NMR spectrum (6a)...4

7-(((1R,5S)-6,6-Dimethylbicyclo[3.1.1]hept-2-en-2-yl)methoxy)-4-methyl-2H-chromen-2-one ^{1}H NMR spectrum (6b) 5

7-(((1S,5R)-6,6-Dimethylbicyclo[3.1.1]hept-2-en-2-yl)methoxy)-4-methyl-2H-chromen-2-one ^{1}H NMR spectrum (6c)....6

7-(2-((1R,5S)-6,6-Dimethylbicyclo[3.1.1]hept-2-en-2-yl)ethoxy)-4-methyl-2H-chromen-2-one ^{1}H NMR spectrum (6d) 7

7-(2-((1R,5S)-6,6-Dimethylbicyclo[3.1.1]hept-2-en-2-yl)ethoxy)-4-methyl-2H-chromen-2-one 13C NMR spectrum (6d) 8

7-(2-((1R,5S)-6,6-Dimethylbicyclo[3.1.1]hept-2-en-2-yl)ethoxy)-4-methyl-2H-chromen-2-one HRMS spectra (6d)....9

(E)-7-(3,7-Dimethylocta-2,6-dienyloxy)-2,3-dihydrocyclopenta[c]chromen-4(1H)-one ^{1}H NMR spectrum (7a) 10

7-(((1R,5S)-6,6-Dimethylbicyclo[3.1.1]hept-2-en-2-yl)methoxy)-2,3-dihydrocyclopenta[c]chromen-4(1H)-one ^{1}H NMR spectrum (7b) 11

7-(((1S,5R)-6,6-Dimethylbicyclo[3.1.1]hept-2-en-2-yl)methoxy)-2,3-dihydrocyclopenta[c]chromen-4(1H)-one ^{1}H NMR spectrum (7c)....12

7-(2-((1R,5S)-6,6-Dimethylbicyclo[3.1.1]hept-2-en-2-yl)ethoxy)-2,3-dihydrocyclopenta[c]chromen-4(1H)-one ^{1}H NMR spectrum (7d) 13

(E)-3-(3,7-Dimethylocta-2,6-dienyloxy)-7,8,9,10-tetrahydro-6H-benzo[c]chromen-6-one ^{1}H NMR spectrum (8a) 14

3-(((1R,5S)-6,6-Dimethylbicyclo[3.1.1]hept-2-en-2-yl)methoxy)-7,8,9,10-tetrahydro-6H-benzo[c]chromen-6-one ^{1}H NMR spectrum (8b) 15

3-(((1S,5R)-6,6-Dimethylbicyclo[3.1.1]hept-2-en-2-yl)methoxy)-7,8,9,10-tetrahydro-6H-benzo[c]chromen-6-one ^{1}H NMR spectrum (8c) 16

3-(2-((1R,5S)-6,6-Dimethylbicyclo[3.1.1]hept-2-en-2-yl)ethoxy)-7,8,9,10-tetrahydro-6H-benzo[c]chromen-6-one ^{1}H NMR spectrum (8d) 17

(E)-7-(3,7-Dimethylocta-2,6-dienyloxy)-4-phenyl-2H-chromen-2-one ^{1}H NMR spectrum (9a) .18

7-(((1R,5S)-6,6-Dimethylbicyclo[3.1.1]hept-2-en-2-yl)methoxy)-4-phenyl-2H-chromen-2-one ^{1}H NMR spectrum (9b) 19

7-(((1S,5R)-6,6-Dimethylbicyclo[3.1.1]hept-2-en-2-yl)methoxy)-4-phenyl-2H-chromen-2-one ^{1}H NMR spectrum (9c)....20

7-(2-((1R,5S)-6,6-dimethylbicyclo[3.1.1]hept-2-en-2-yl)ethoxy)-4-phenyl-2H-chromen-2-one ^{1}H NMR spectrum (9d)....21

(E)-7-(3,7-Dimethylocta-2,6-dienyloxy)-4-(4-fluorophenyl)-2H-chromen-2-one ^{1}H NMR spectrum (10a) ... 22

7-(((1R,5S)-6,6-Dimethylbicyclo[3.1.1]hept-2-en-2-yl)methoxy)-4-(4-fluorophenyl)-2H-chromen-2-one ^{1}H NMR spectrum (10b) ... 23

7-(((1S,5R)-6,6-Dimethylbicyclo[3.1.1]hept-2-en-2-yl)methoxy)-4-(4-fluorophenyl)-2H-chromen-2-one ^{1}H NMR spectrum (10c) ... 24

7-(2-((1R,5S)-6,6-Dimethylbicyclo[3.1.1]hept-2-en-2-yl)ethoxy)-4-(4-fluorophenyl)-2H-chromen-2-one ^{1}H NMR spectrum (10d) ... 25

Results of the OGTT-1 ... 26

Results of the OGTT-2 ... 26

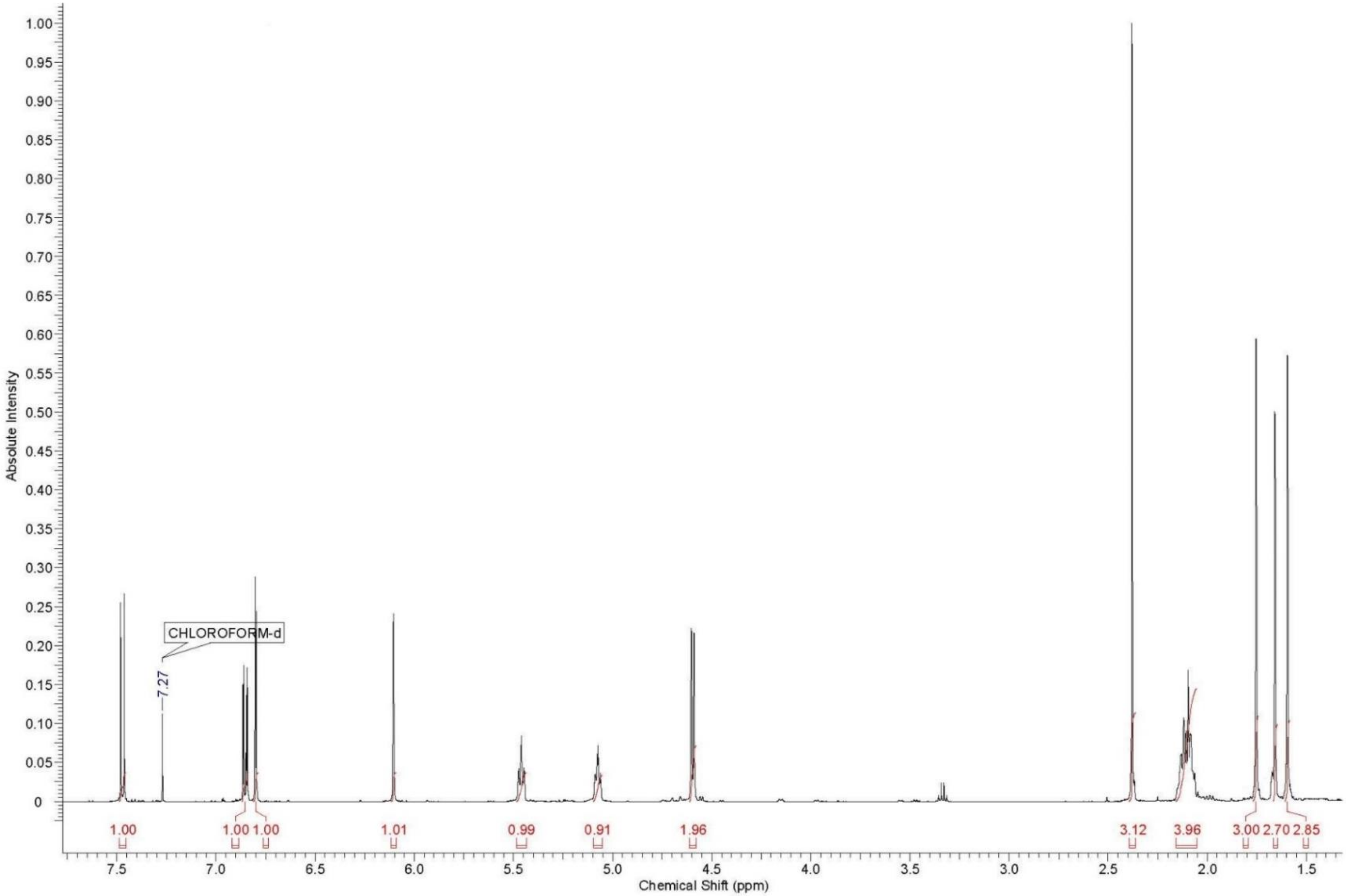

Figure S1. (E)-7-(3,7-Dimethylocta-2,6-dienyloxy)-4-methyl-2H-chromen-2-one ^{1}H NMR spectrum (6a).

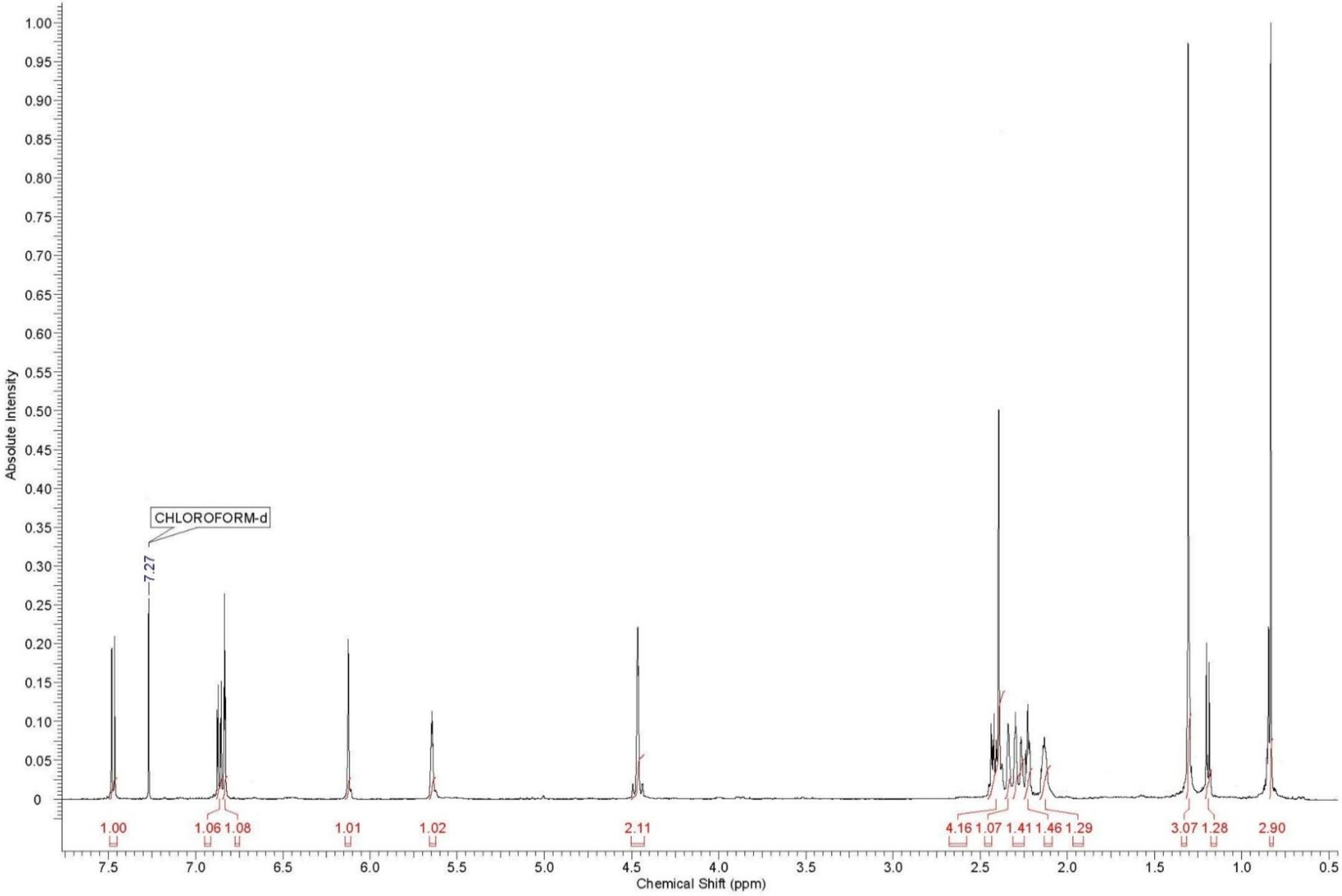

Figure S2. 7-(((1R,5S)-6,6-Dimethylbicyclo[3.1.1]hept-2-en-2-yl)methoxy)-4-methyl-2H-chromen-2-one ^{1}H NMR spectrum (6b).

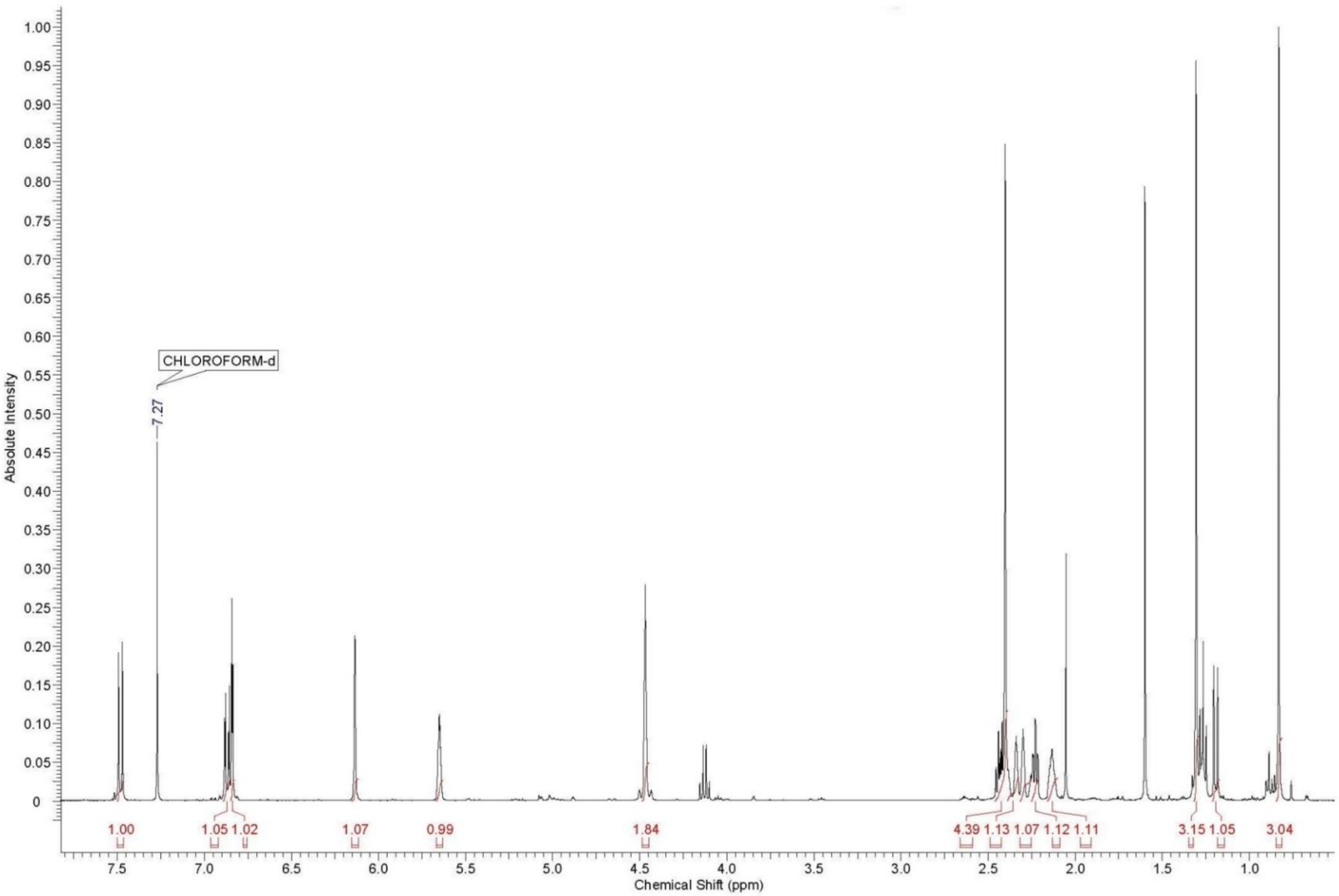

Figure S3. 7-(((1S,5R)-6,6-Dimethylbicyclo[3.1.1]hept-2-en-2-yl)methoxy)-4-methyl-2H-chromen-2-one 1H NMR spectrum (6c).

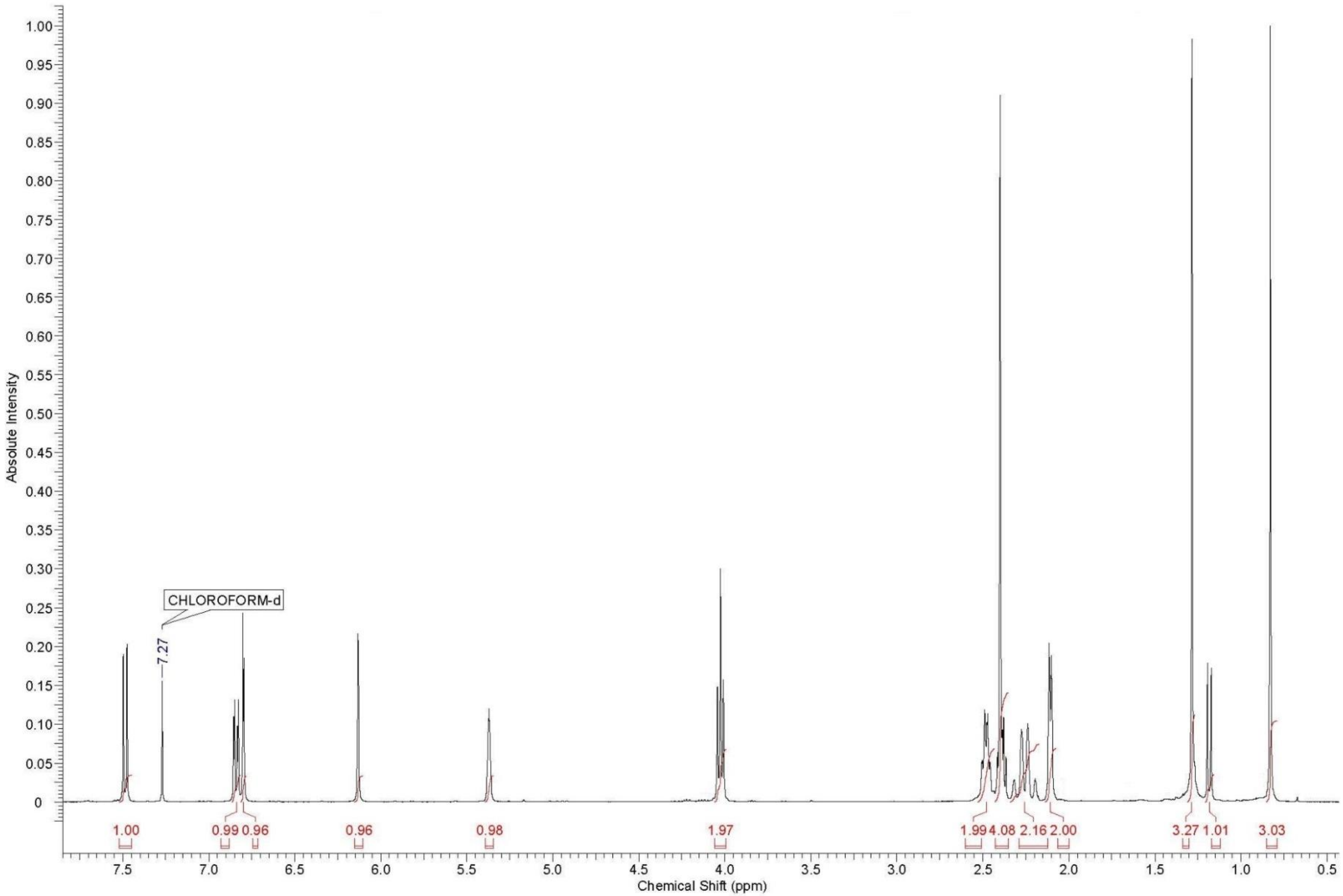

Figure S4. 7-(2-((1R,5S)-6,6-Dimethylbicyclo[3.1.1]hept-2-en-2-yl)ethoxy)-4-methyl-2H-chromen-2-one 1H NMR spectrum (6d).

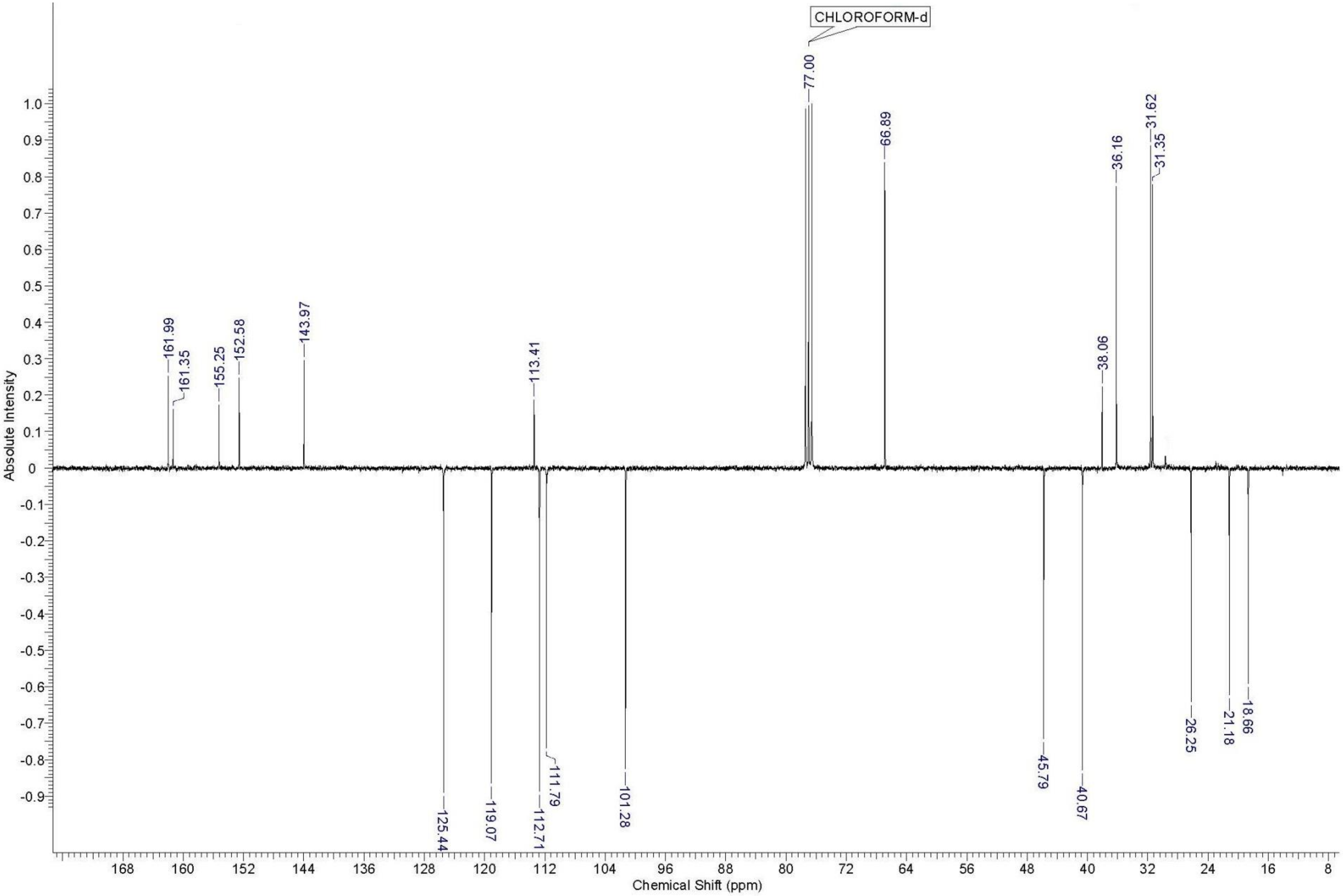

Figure S5. 7-(2-((1R,5S)-6,6-Dimethylbicyclo[3.1.1]hept-2-en-2-yl)ethoxy)-4-methyl-2H-chromen-2-one 13C NMR spectrum (6d).

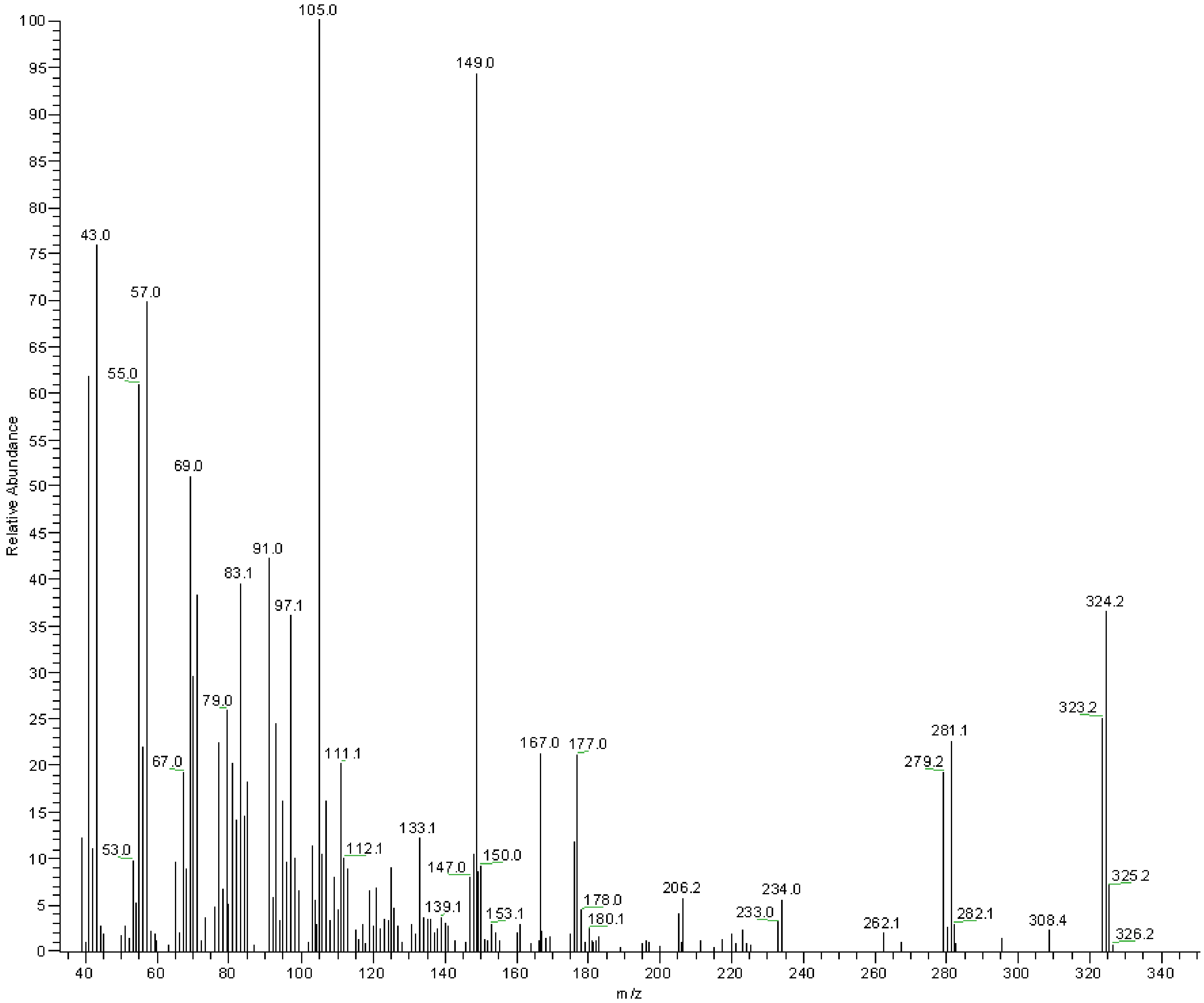

Figure S6. 7-(2-((1R,5S)-6,6-Dimethylbicyclo[3.1.1]hept-2-en-2-yl)ethoxy)-4-methyl-2H-chromen-2-one HRMS spectra (6d).

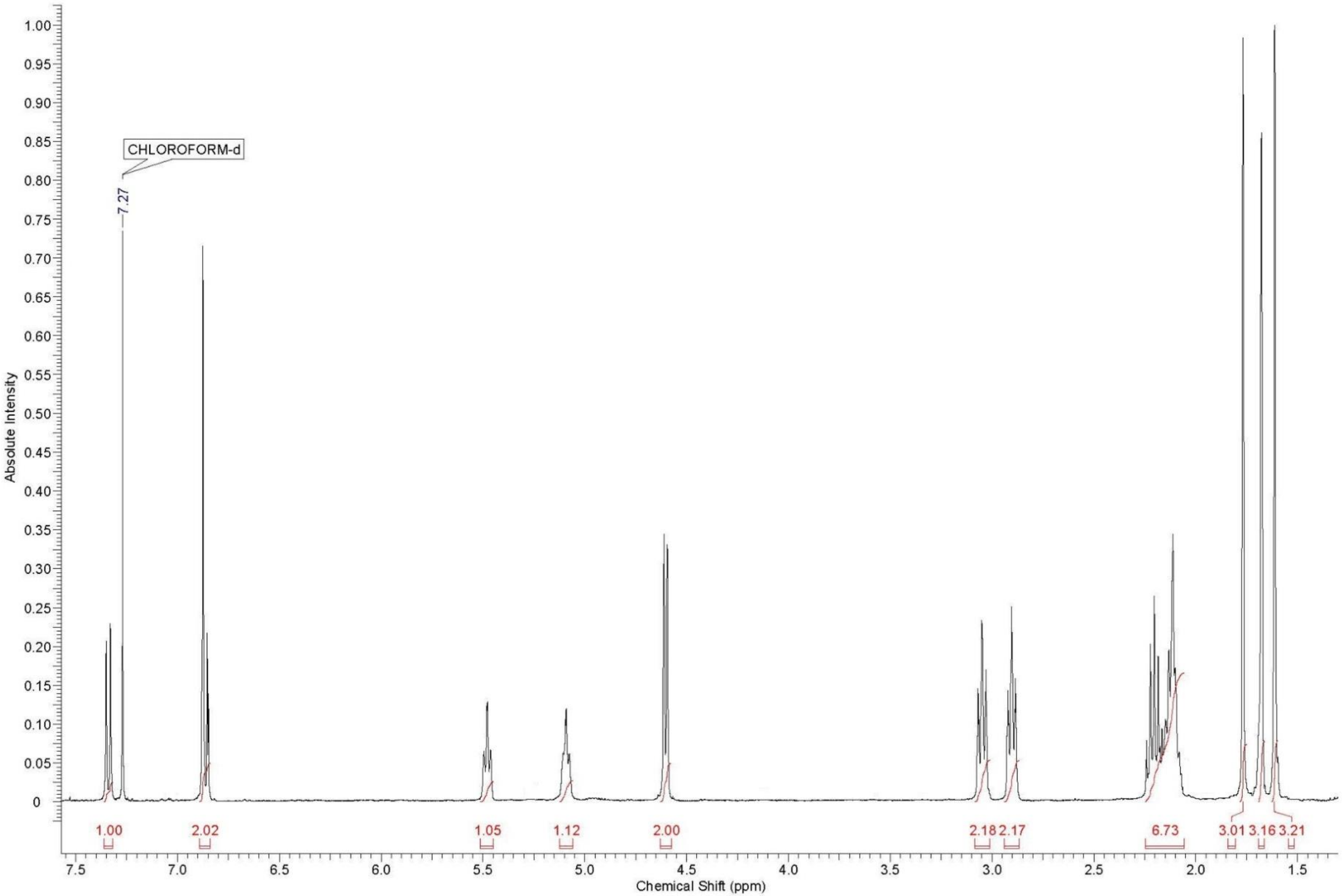

Figure S7. (E)-7-(3,7-Dimethylocta-2,6-dienyloxy)-2,3-dihydrocyclopenta[c]chromen-4(1H)-one 1H NMR spectrum (7a).

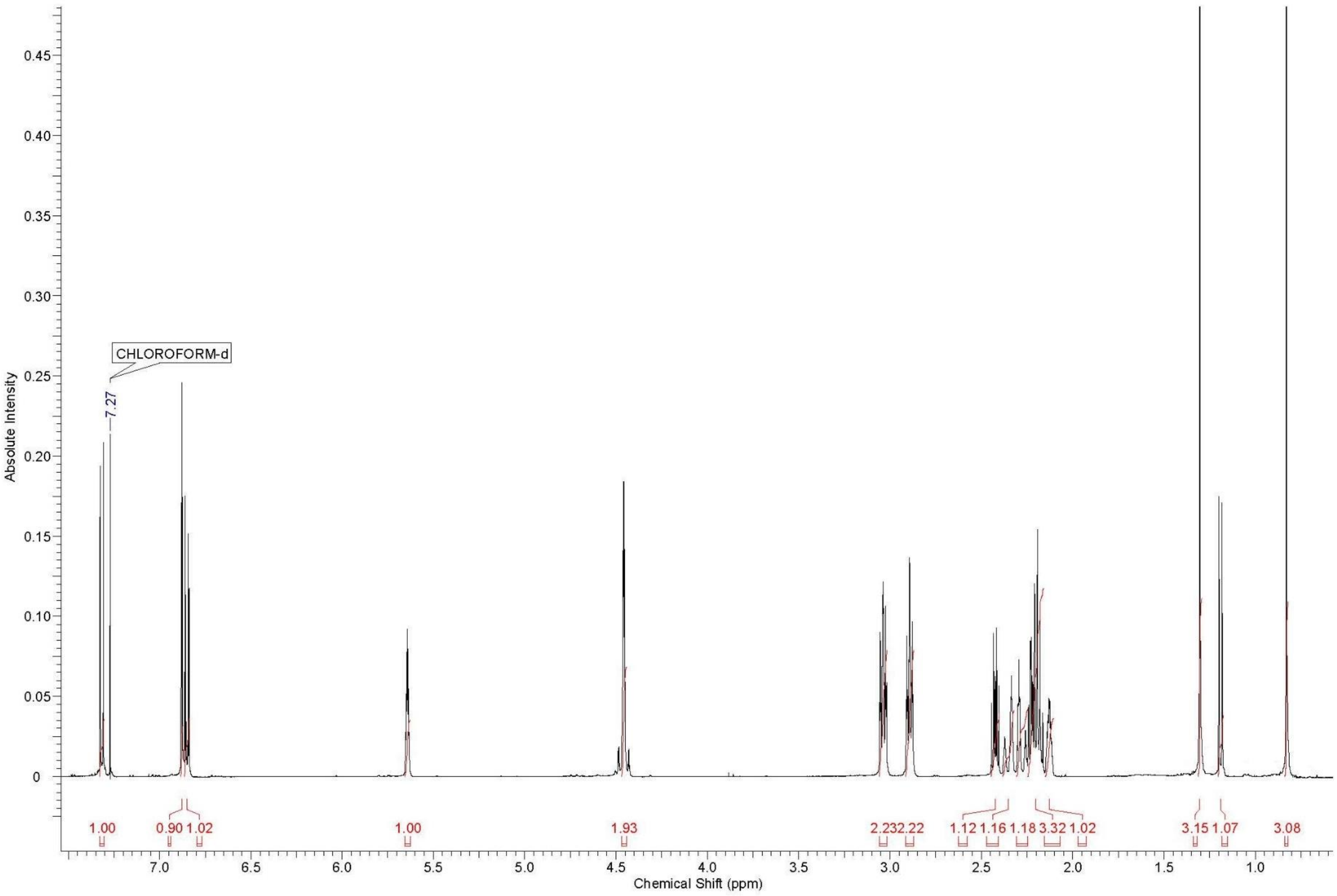

Figure S8. 7-(((1R,5S)-6,6-Dimethylbicyclo[3.1.1]hept-2-en-2-yl)methoxy)-2,3-dihydrocyclopenta[c]chromen-4(1H)-one 1H NMR spectrum (7b).

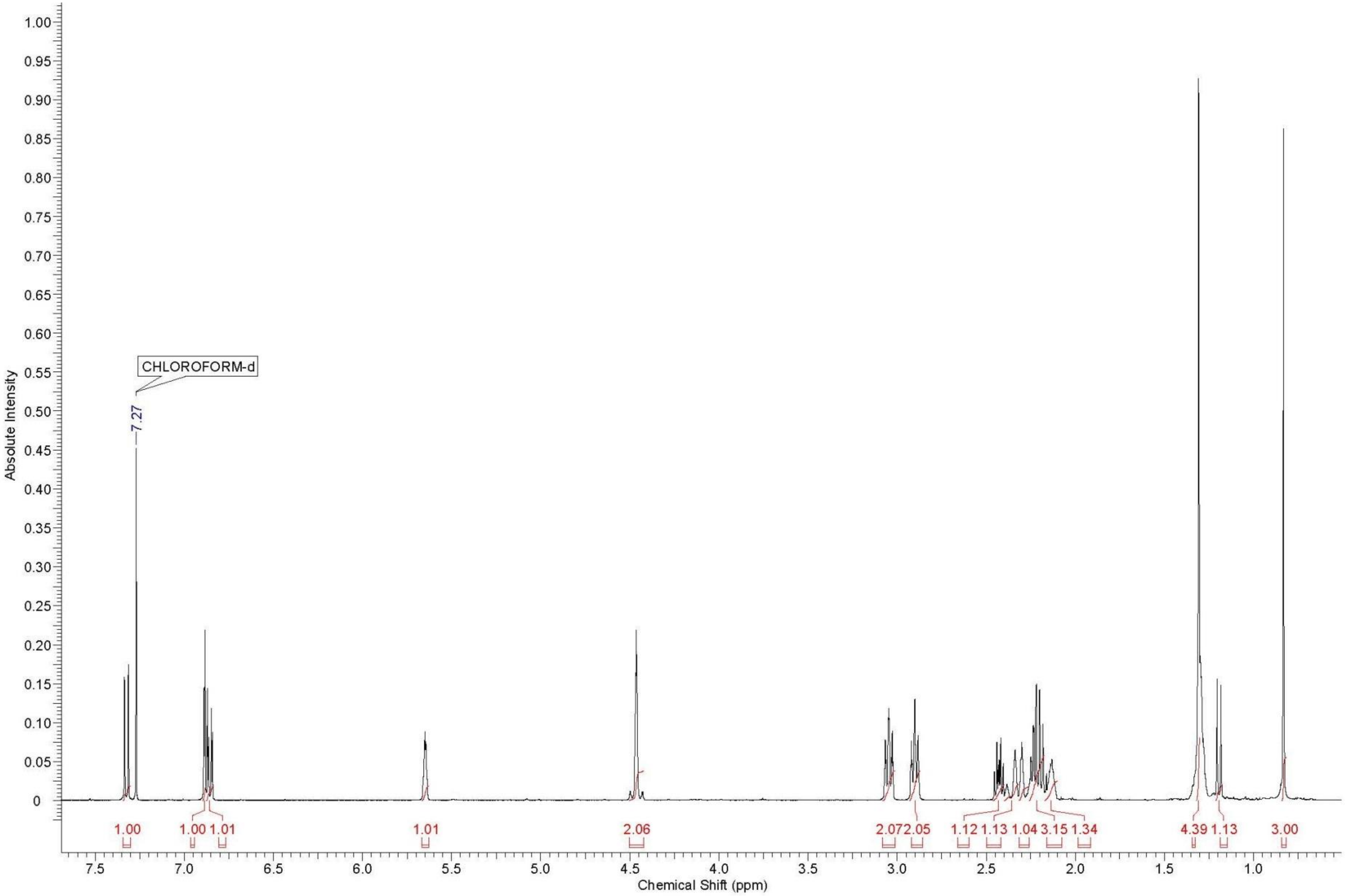

Figure S9. 7-(((1S,5R)-6,6-Dimethylbicyclo[3.1.1]hept-2-en-2-yl)methoxy)-2,3-dihydrocyclopenta[c]chromen-4(1H)-one ^{1}H NMR spectrum (7c).

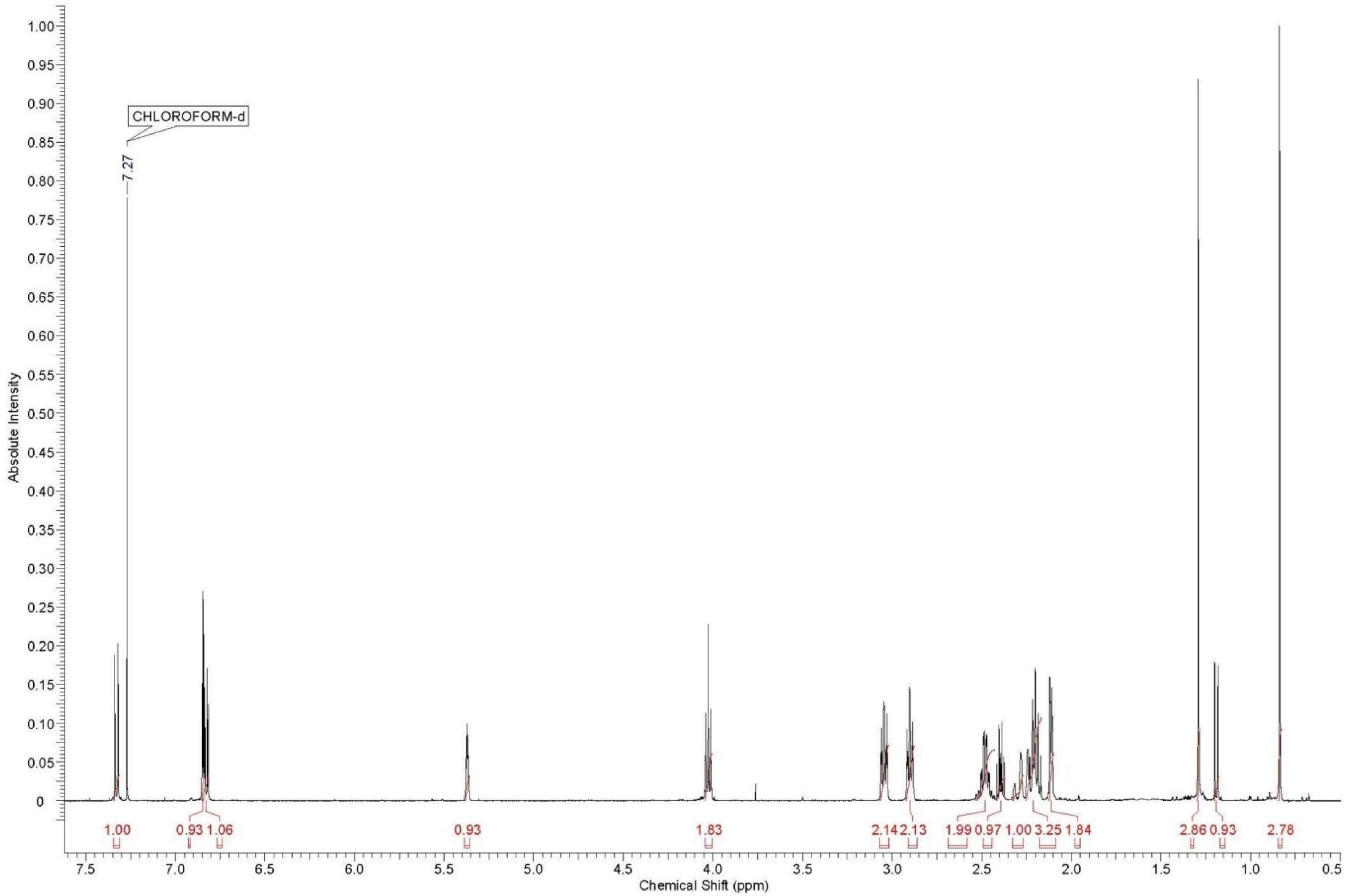

Figure S10. 7-(2-((1R,5S)-6,6-Dimethylbicyclo[3.1.1]hept-2-en-2-yl)ethoxy)-2,3-dihydrocyclopenta[c]chromen-4(1H)-one ^{1}H NMR spectrum (7d).

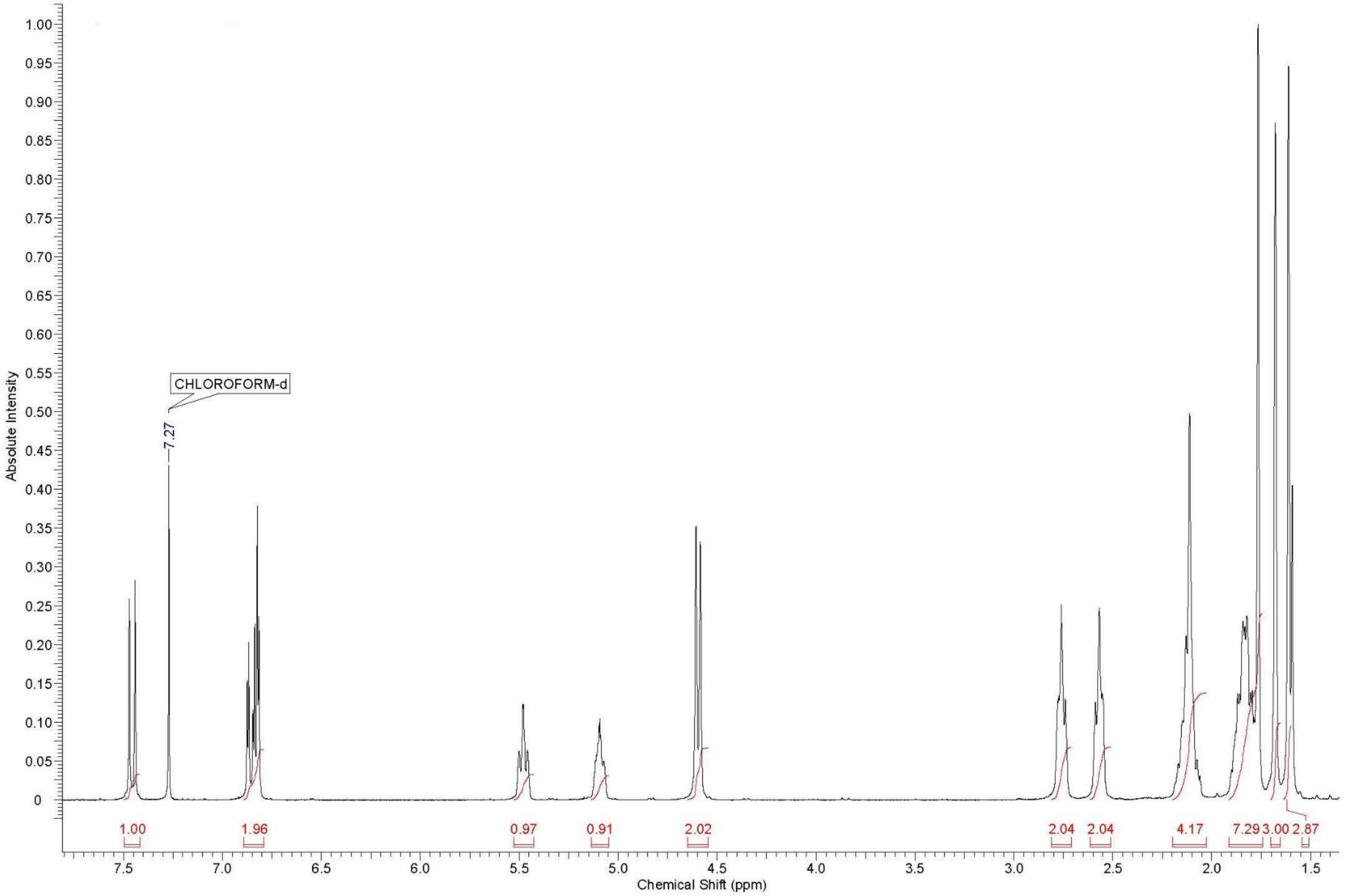

Figure S11. (E)-3-(3,7-Dimethylocta-2,6-dienyloxy)-7,8,9,10-tetrahydro-6H-benzo[c]chromen-6-one ^{1}H NMR spectrum (8a).

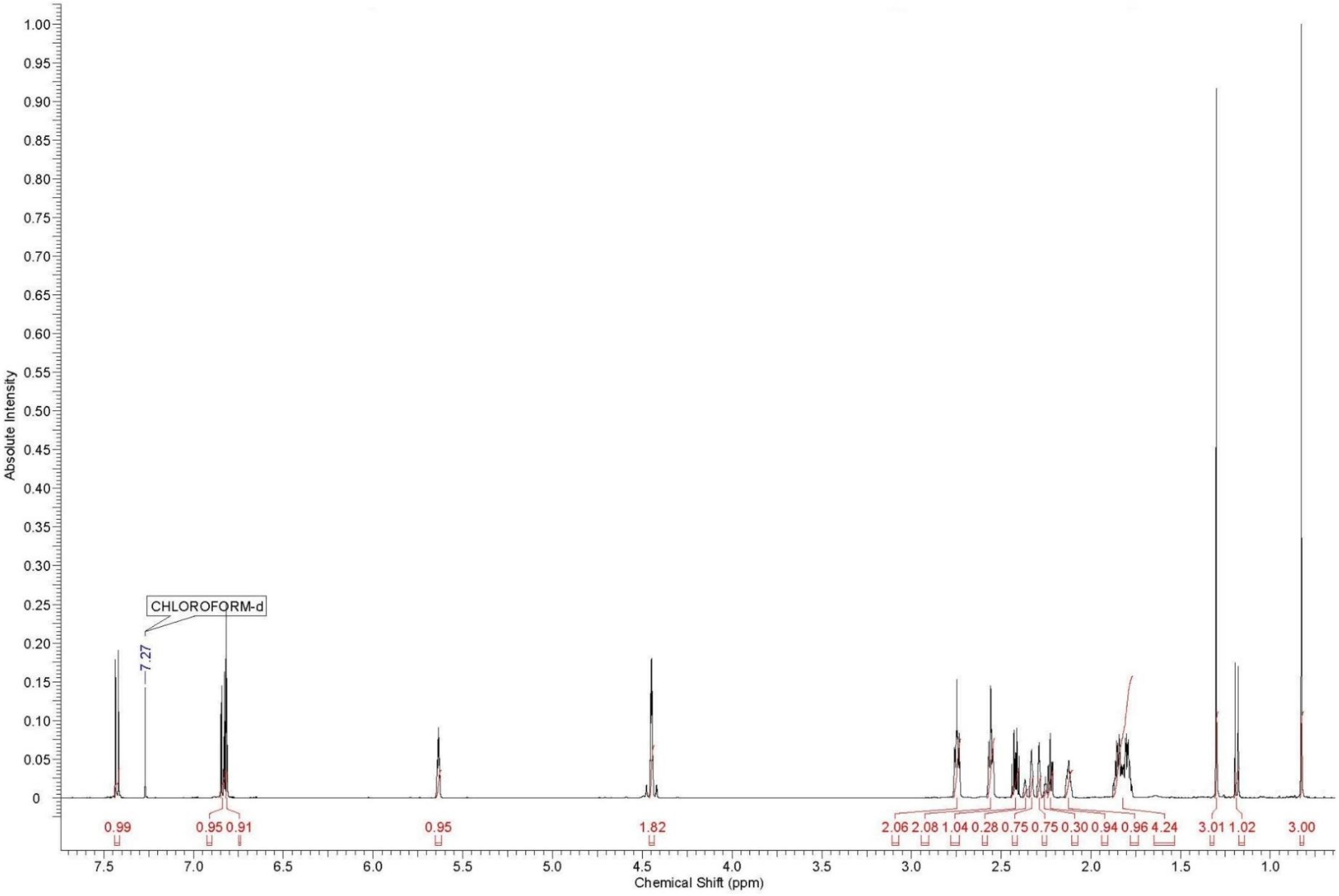

Figure S12. 3-(((1R,5S)-6,6-Dimethylbicyclo[3.1.1]hept-2-en-2-yl)methoxy)-7,8,9,10-tetrahydro-6H-benzo[c]chromen-6-one ^{1}H NMR spectrum (8b).

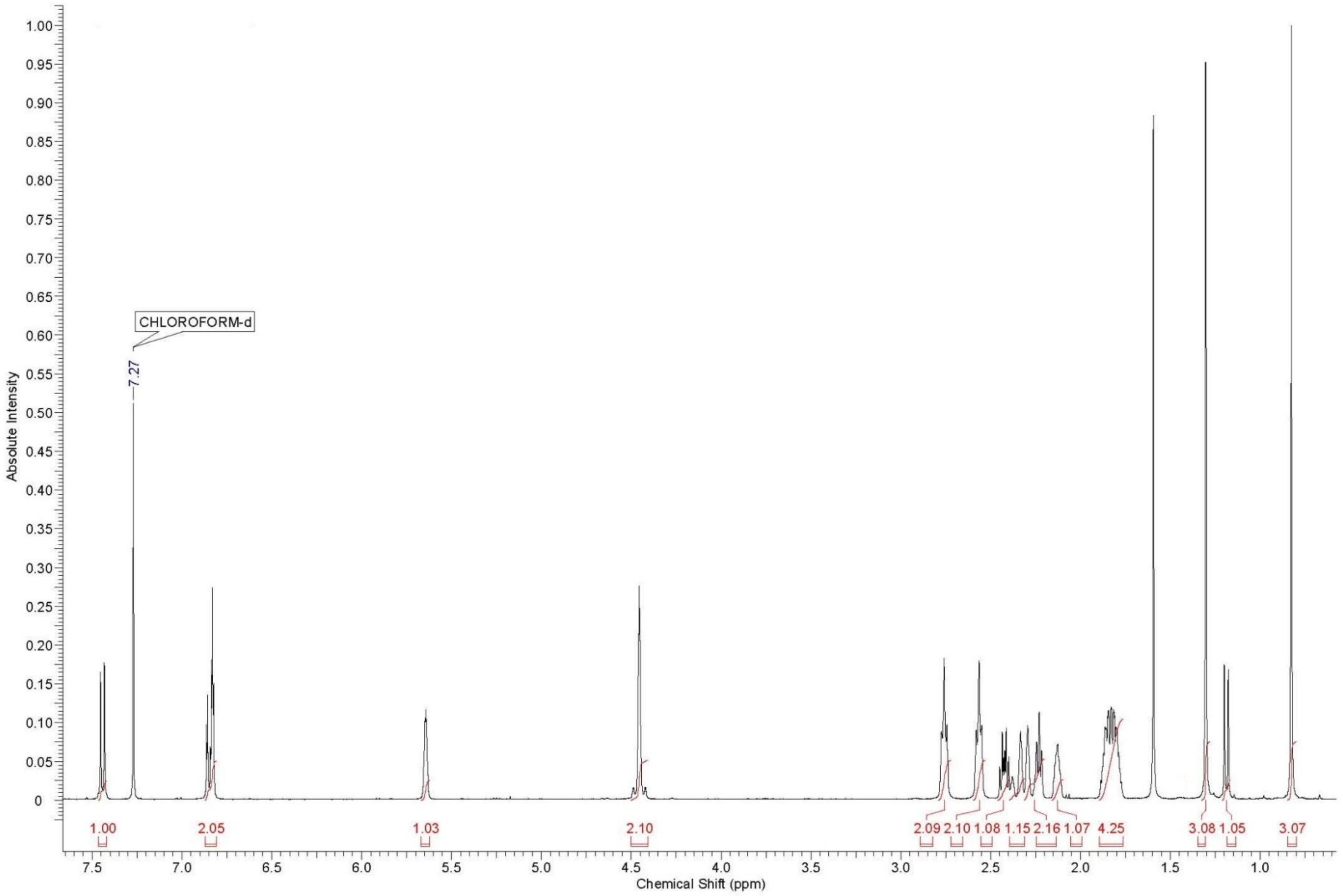

Figure S13. 3-(((1S,5R)-6,6-Dimethylbicyclo[3.1.1]hept-2-en-2-yl)methoxy)-7,8,9,10-tetrahydro-6H-benzo[c]chromen-6-one ^{1}H NMR spectrum (8c).

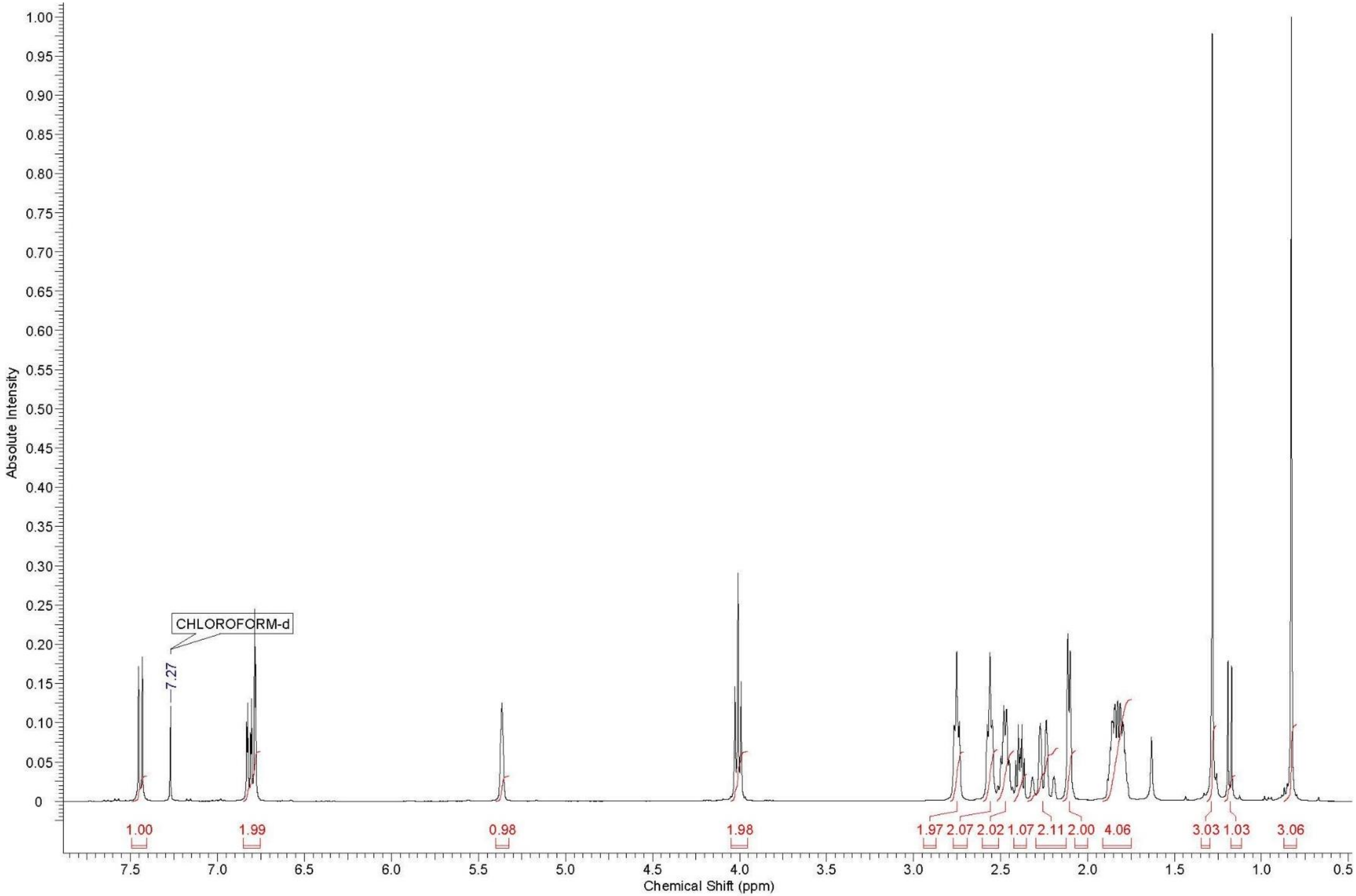

Figure S14. 3-(2-((1R,5S)-6,6-Dimethylbicyclo[3.1.1]hept-2-en-2-yl)ethoxy)-7,8,9,10-tetrahydro-6H-benzo[c]chromen-6-one ^{1}H NMR spectrum (8d).

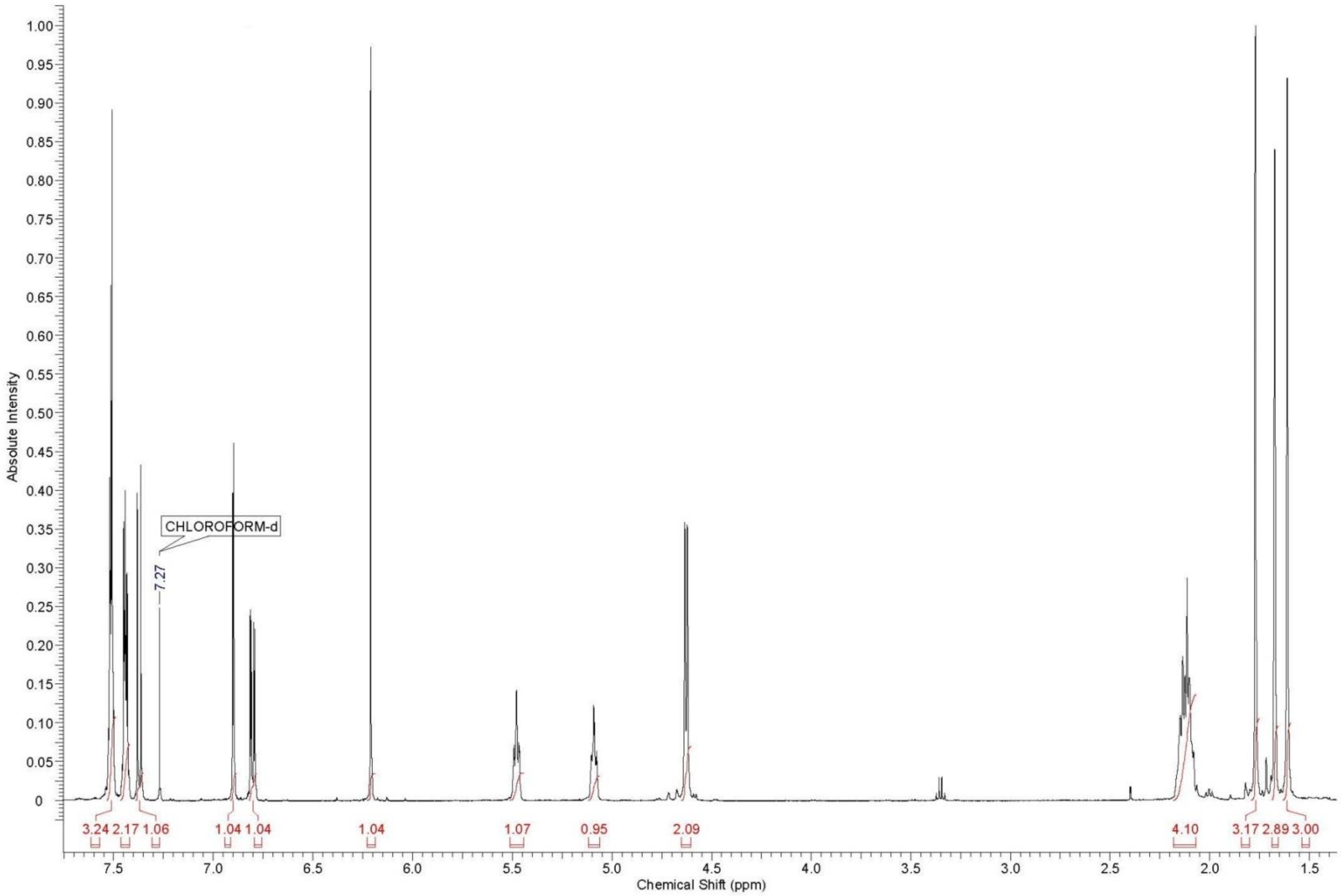

Figure S15. (E)-7-(3,7-Dimethylocta-2,6-dienyloxy)-4-phenyl-2H-chromen-2-one ^{1}H NMR spectrum (9a).

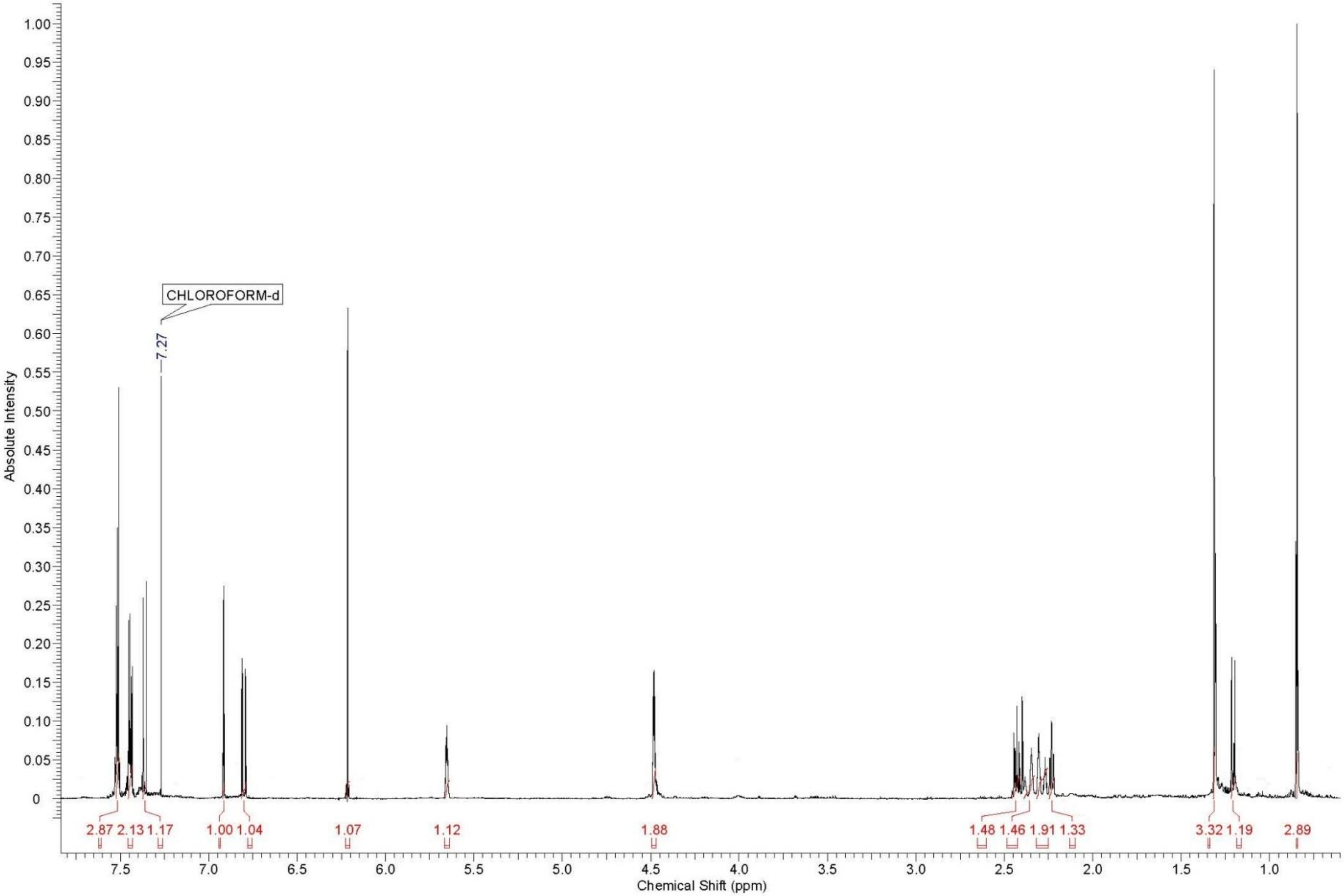

Figure S16. 7-(((1R,5S)-6,6-Dimethylbicyclo[3.1.1]hept-2-en-2-yl)methoxy)-4-phenyl-2H-chromen-2-one ^{1}H NMR spectrum (9b).

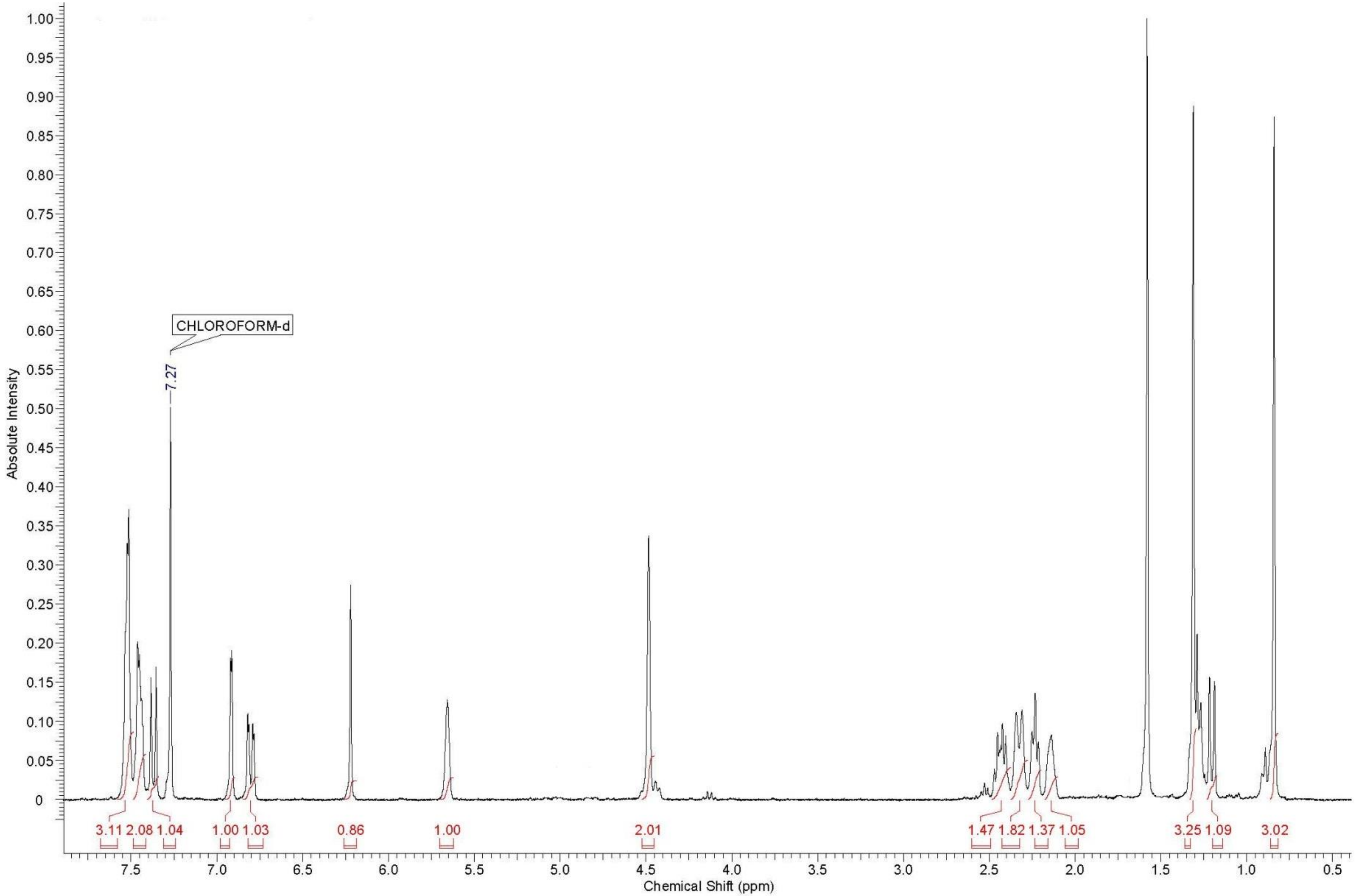

Figure S17. 7-(((1S,5R)-6,6-Dimethylbicyclo[3.1.1]hept-2-en-2-yl)methoxy)-4-phenyl-2H-chromen-2-one ^{1}H NMR spectrum (9c).

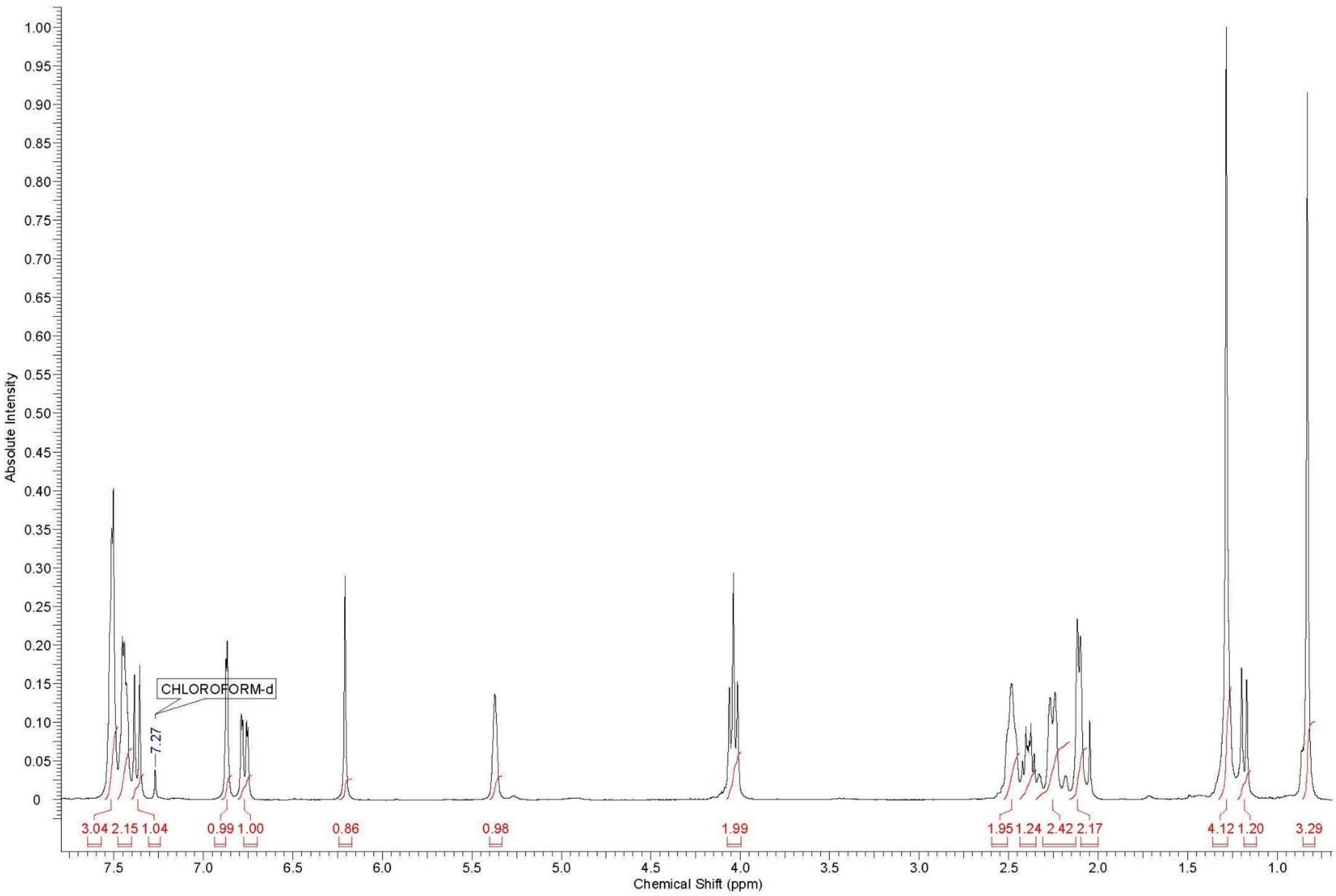

Figure S18. 7-(2-((1R,5S)-6,6-dimethylbicyclo[3.1.1]hept-2-en-2-yl)ethoxy)-4-phenyl-2H-chromen-2-one ^{1}H NMR spectrum (9d).

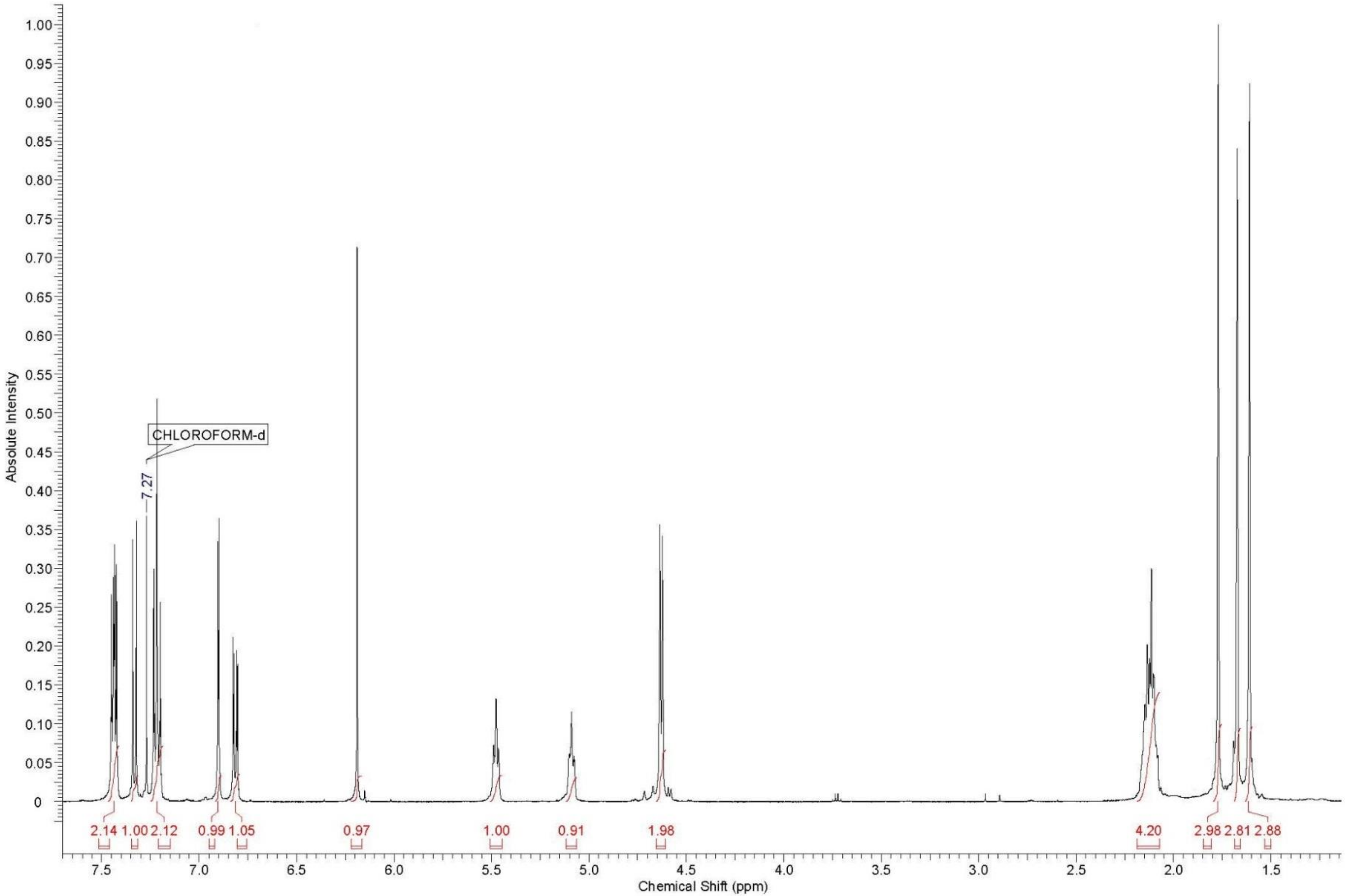

Figure S19. (E)-7-(3,7-Dimethylocta-2,6-dienyloxy)-4-(4-fluorophenyl)-2H-chromen-2-one ^{1}H NMR spectrum (10a).

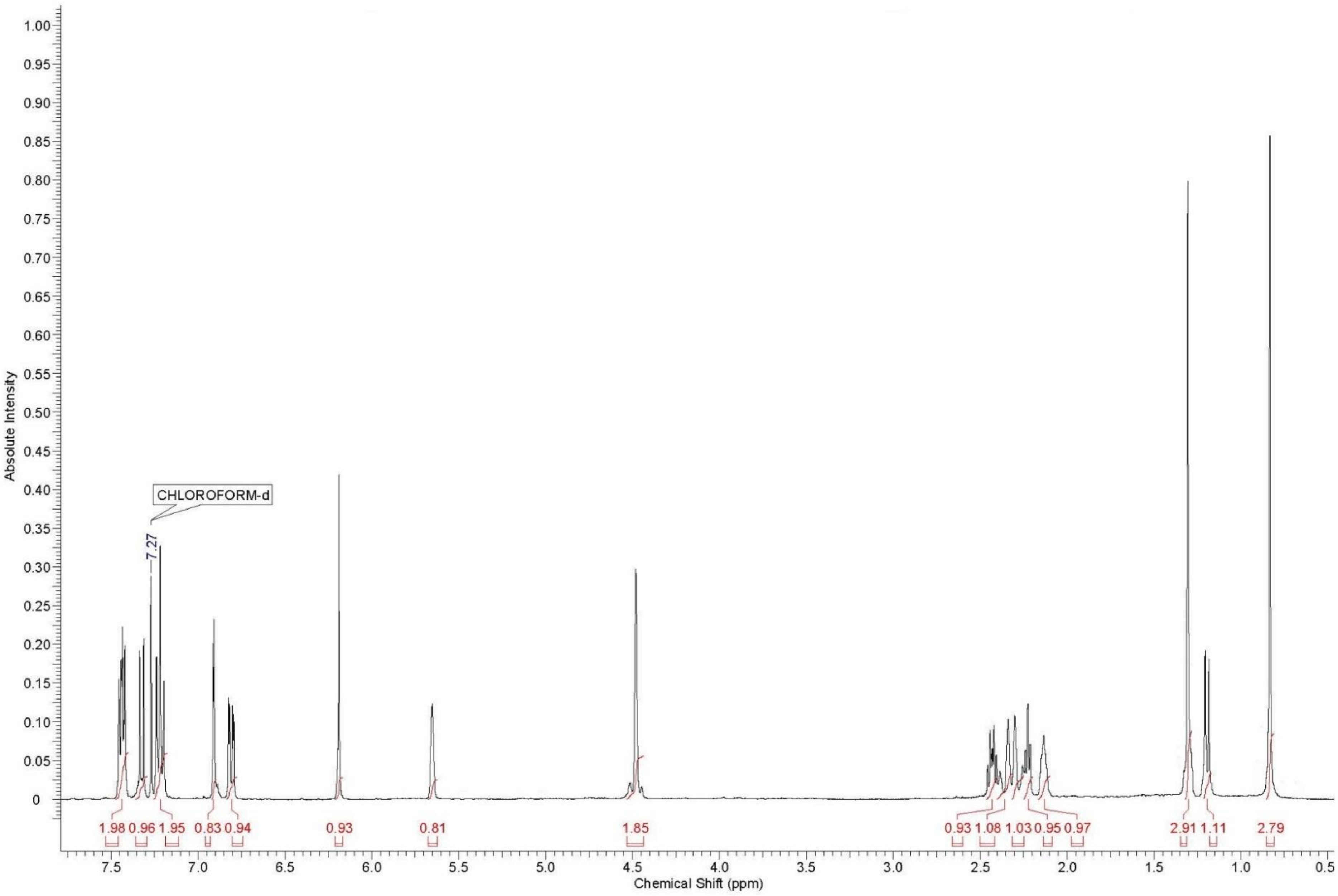

Figure S20. 7-(((1R,5S)-6,6-Dimethylbicyclo[3.1.1]hept-2-en-2-yl)methoxy)-4-(4-fluorophenyl)-2H-chromen-2-one ^{1}H NMR spectrum (10b).

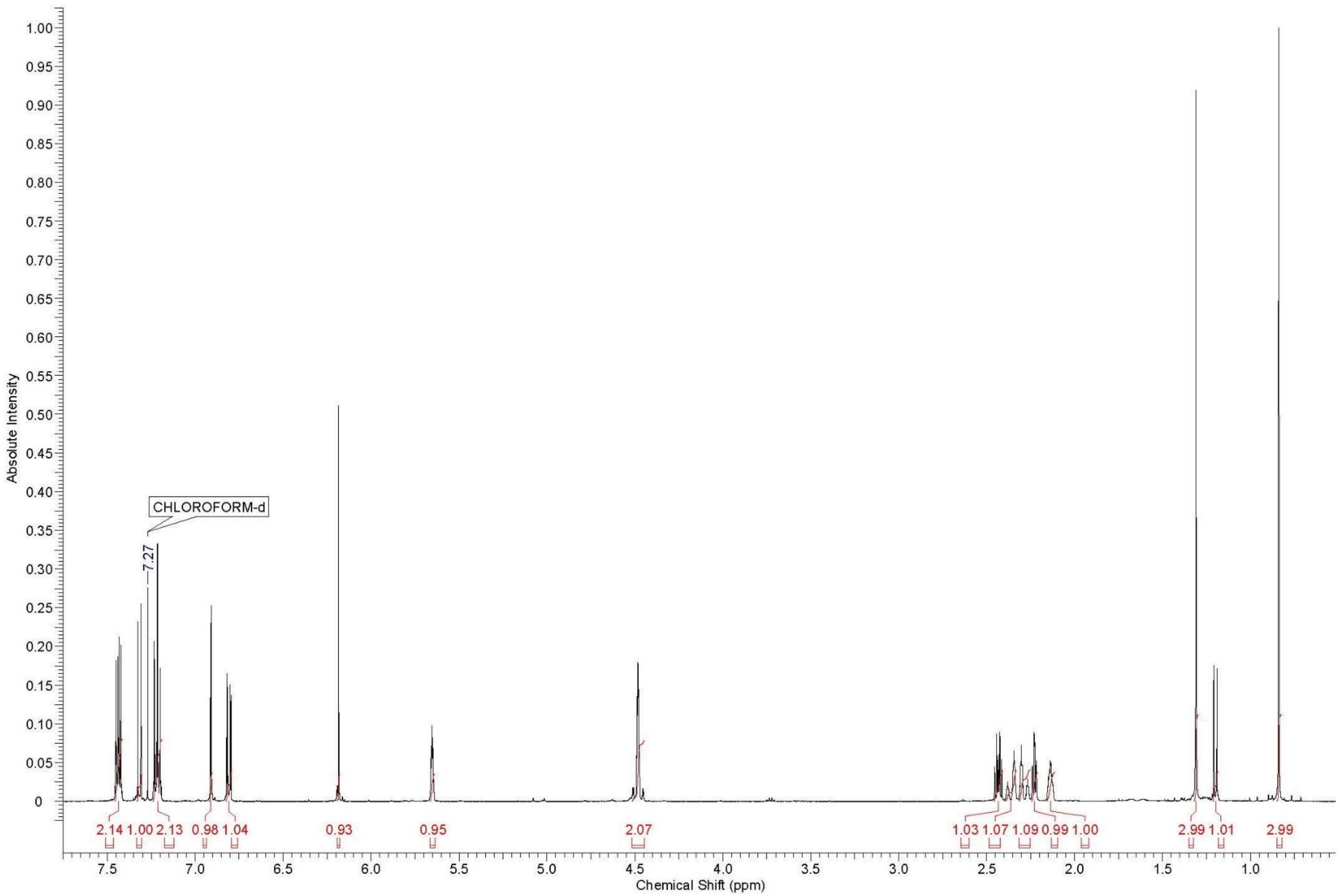

Figure S21. 7-(((1S,5R)-6,6-Dimethylbicyclo[3.1.1]hept-2-en-2-yl)methoxy)-4-(4-fluorophenyl)-2H-chromen-2-one ^{1}H NMR spectrum (10c).

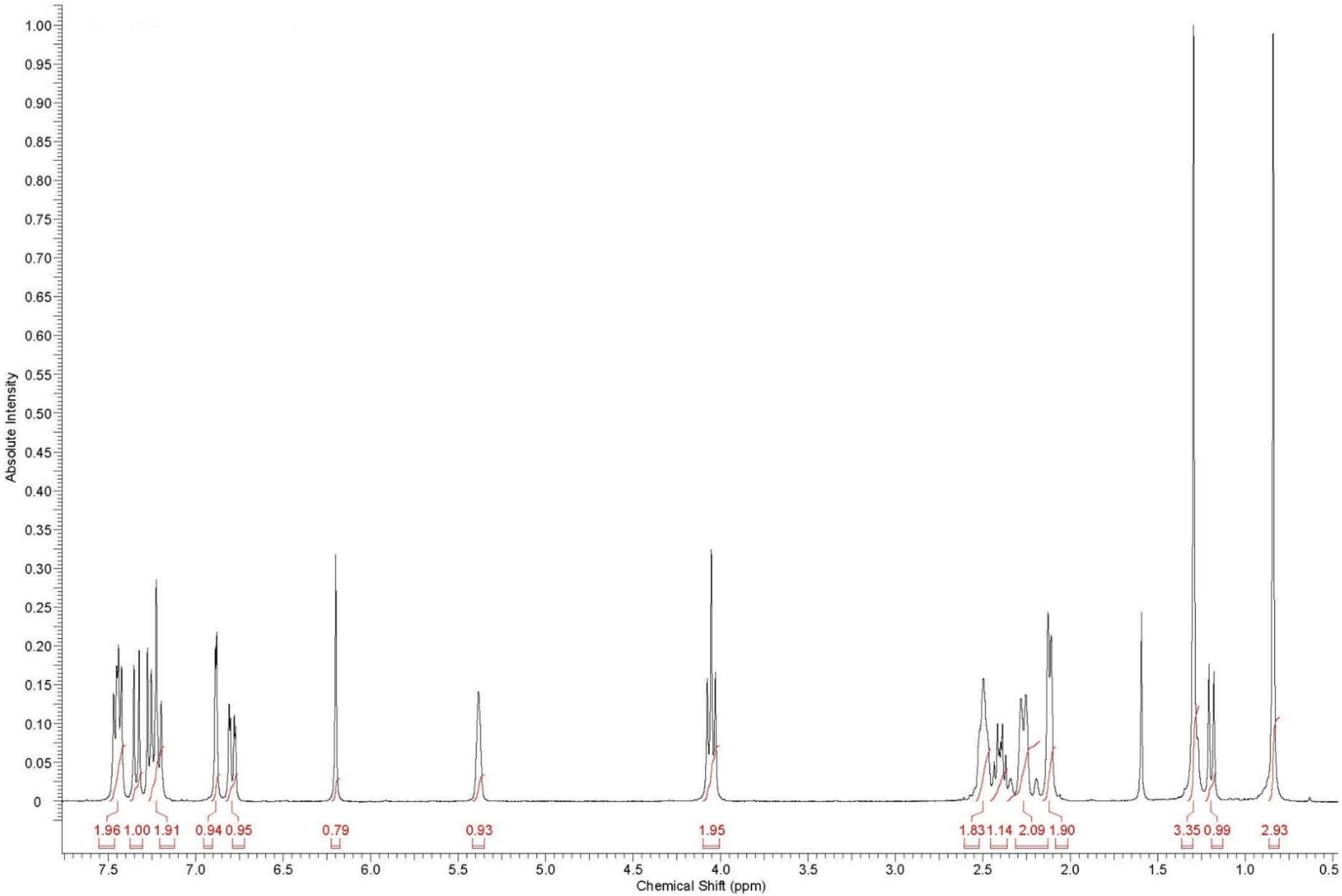

Figure S22. 7-(2-((1R,5S)-6,6-Dimethylbicyclo[3.1.1]hept-2-en-2-yl)ethoxy)-4-(4-fluorophenyl)-2H-chromen-2-one ^{1}H NMR spectrum (10d).

Results of the OGTT-1.

	AUC (Glycemic curve)	SEM
Control	1175,5	27,90
Vildagliptin, 10 mg/kg	748,5	18,22
9a, 15 mg/kg	1048,3	40,96
9c, 15 mg/kg	1376,0	26,29
10b, 15 mg/kg	1346,0	61,21

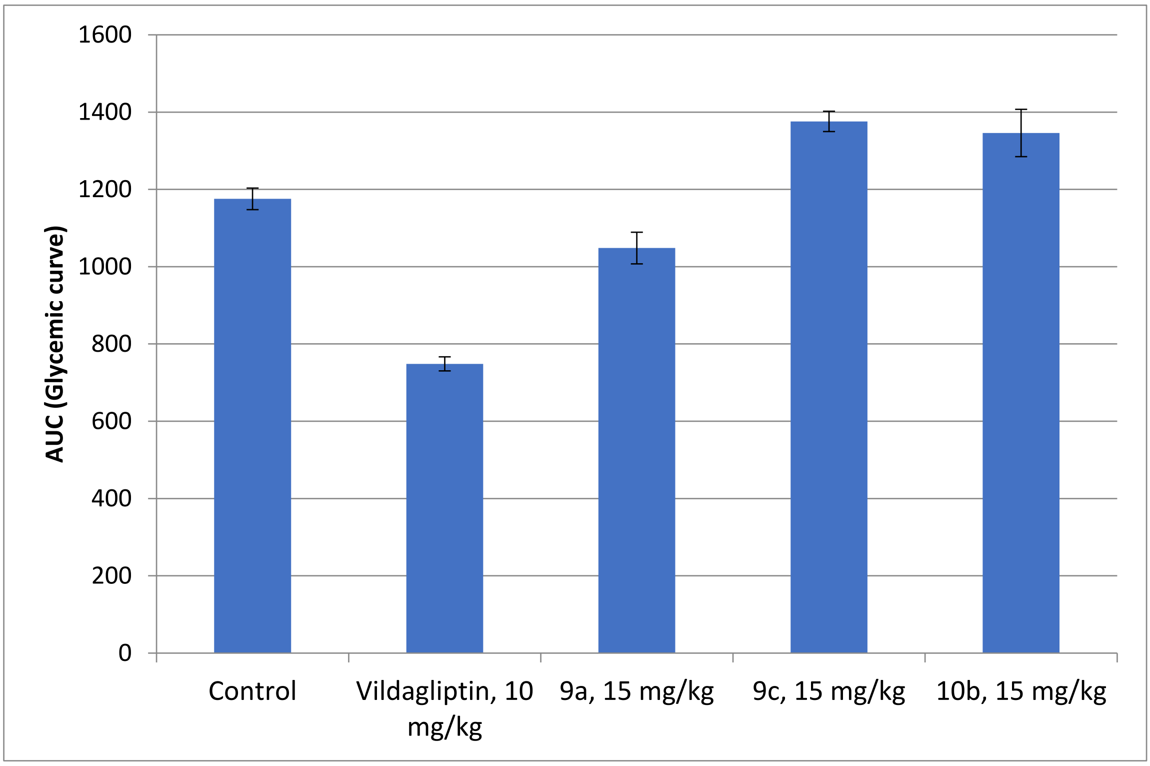

Figure S23. Data is presented as the area under the glycemic curve (AUC). *p≤0,05 lower compared to the control group; #p≤0,05 higher compared to the control group (higher).

Results of the OGTT-2.

	AUC (Glycemic curve)	SEM
Control	1736,0	32,08
Vildagliptin, 10 mg/kg	1134,8	49,61
7b, 10 mg/kg	1513,0	54,37
10c, 15 mg/kg	1664,0	32,48

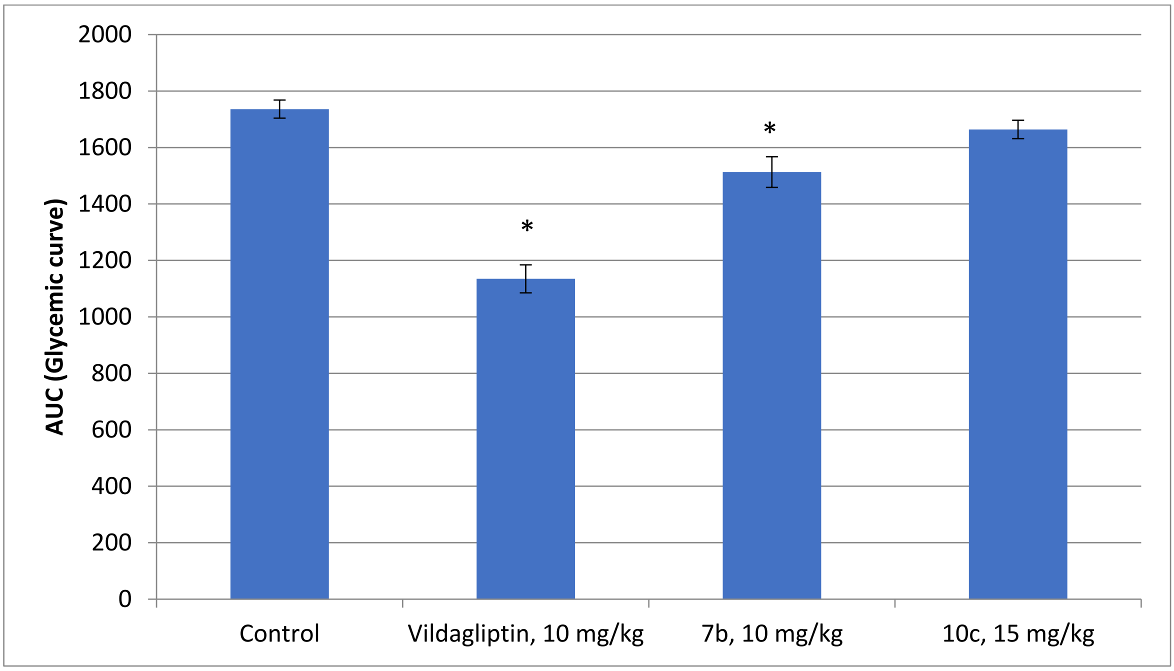

Figure S24. lower compared to the control group; #p≤0,05 higher compared to the control group (higher).
